# ADAPT: Analysis of Microbiome Differential Abundance by Pooling Tobit Models

**DOI:** 10.1093/bioinformatics/btae661

**Published:** 2024-11-07

**Authors:** Mukai Wang, Simon Fontaine, Hui Jiang, Gen Li

**Affiliations:** Department of Biostatistics, University of Michigan, 1415 Washington Heights, Ann Arbor, Michigan, 48109, United States; Department of Statistics, University of Michigan, 1085 South University, Ann Arbor, Michigan, 48109, United States; Department of Biostatistics, University of Michigan, 1415 Washington Heights, Ann Arbor, Michigan, 48109, United States; Department of Biostatistics, University of Michigan, 1415 Washington Heights, Ann Arbor, Michigan, 48109, United States

## Abstract

**Motivation:**

Microbiome differential abundance analysis (DAA) remains a challenging problem despite multiple methods proposed in the literature. The excessive zeros and compositionality of metagenomics data are two main challenges for DAA.

**Results:**

We propose a novel method called “Analysis of Microbiome Differential Abundance by Pooling Tobit Models” (ADAPT) to overcome these two challenges. ADAPT interprets zero counts as left-censored observations to avoid unfounded assumptions and complex models. ADAPT also encompasses a theoretically justified way of selecting non-differentially abundant microbiome taxa as a reference to reveal differentially abundant taxa while avoiding false discoveries. We generate synthetic data using independent simulation frameworks to show that ADAPT has more consistent false discovery rate control and higher statistical power than competitors. We use ADAPT to analyze 16S rRNA sequencing of saliva samples and shotgun metagenomics sequencing of plaque samples collected from infants in the COHRA2 study. The results provide novel insights into the association between the oral microbiome and early childhood dental caries.

**Availability and implementation:**

The R package ADAPT can be installed from Bioconductor at https://bioconductor.org/packages/release/bioc/html/ADAPT.html or from Github at https://github.com/mkbwang/ADAPT. The source codes for simulation studies and real data analysis are available at https://github.com/mkbwang/ADAPT_example.

## 1 Introduction

The microbiome plays an essential role in human health and disease. Extensive research has been conducted into the human microbiome using high-throughput metagenomic sequencing technologies (Human Microbiome Project Consortium 2012, Yatsunenko *et al.* 2012, McDonald *et al.* 2018). Metagenomics data consist of count tables that represent each sample’s abundance profiles of microbiome taxa. Differential abundance analysis (DAA) identifies taxa whose abundances differ between conditions. This is one of the fundamental analyses of microbiome data (Li 2015). Many methods have been proposed to tackle statistical challenges in identifying differentially abundant (DA) taxa. However, no solution has been shown to be universally preferable (Nearing *et al.* 2022, Yang and Chen 2022).

**Figure 1.**
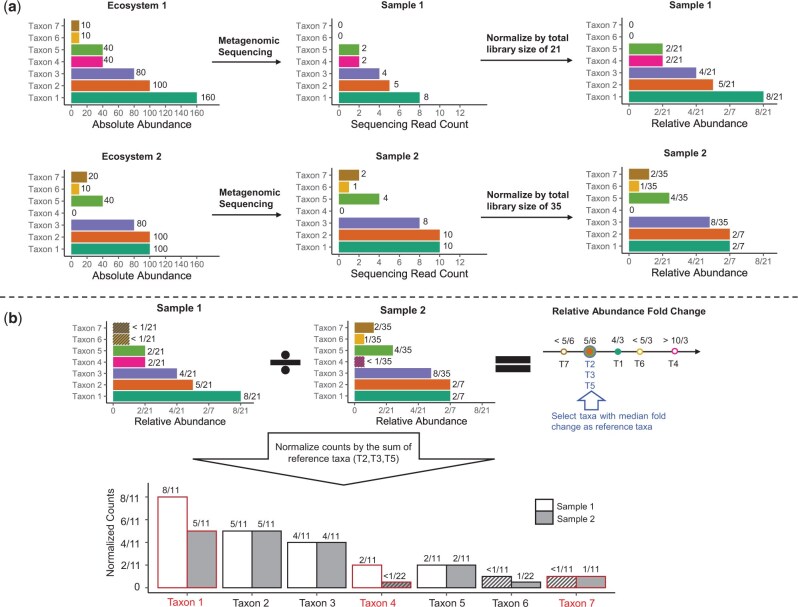
Illustration of ADAPT with a toy example. **(a)** Three microbiome taxa (taxa 1, 4, and 7) are DA between two ecosystems. Neither the observed counts nor the relative abundances can be directly compared for DAA. **(b)** ADAPT treats zero counts as left-censored at the detection limit (one in this case). ADAPT first calculates the fold change of relative abundances. It then selects a subset of taxa (taxa 2, 3, and 5) whose fold changes equal the median as reference taxa. After scaling the counts by the sum of three reference taxa, ADAPT can recover the DA taxa without false positives by comparing the normalized counts.

Metagenomics count data have excessive zeros (Kaul *et al.* 2017, Silverman *et al.* 2020). As illustrated in the toy example in Fig. 1a, zeros might reflect the actual absence of taxa in one condition (biological zeros) or indicate rare taxa that the sequencing procedure did not detect (sampling zeros). Some DAA methods impute the zeros with a small positive constant, often called “pseudo-counts” (Mandal *et al.* 2015, Lin and Peddada 2020, 2024, Mallick *et al.* 2021, Zhou *et al.* 2022). The imputations pave the way for applying standard statistical models to log counts. However, these imputations assume that all zeros are sampling zeros and ignore that library sizes vary among samples, which may lead to inflated false discovery rates (Nearing *et al.* 2022, Yang and Chen 2022). Other methods fit statistical distributions to the counts or count proportions and then draw from the fitted distributions to retrieve smoothed counts and count proportions for downstream analysis (Fernandes *et al.* 2014, Brill *et al.* 2022, Yang and Chen 2022). These methods have better control of false discovery rates (Yang and Chen 2022), but their distribution choices lack justification. A third group of methods adopts zero-inflated distributions (Paulson *et al.* 2013, Sohn *et al.* 2015). Zero-inflated distributions agree with the sparsity patterns in metagenomics data. However, fitting a zero-inflated distribution involves estimating the probability of true zeros and the distribution parameters for nonzero values. Combining two hypothesis tests for DAA reduces power and inflates false discovery rates.

Metagenomics data are also compositional (Gloor *et al.* 2017). As illustrated in Fig. 1a, the metagenomics sequencing read counts are not directly comparable between samples because of varying sequencing depths. The counts can be interpretable after they are scaled by the library sizes and transformed into relative abundances. However, taxa may have different relative abundances between conditions, while their absolute abundances remain stable. The key to DAA is to find an appropriate scaling factor to bridge the gap between relative and absolute abundances. Some methods normalize the counts with centered log-ratio (CLR) transformation (Fernandes *et al.* 2014). The CLR transformation uses the geometric mean of all taxa counts as the scaling factor. The geometric mean calculation involves DA taxa, which could yield false positives. Other methods derive bias correction factors and have them multiplied with relative abundances or added to CLR-transformed counts (Paulson *et al.* 2013, Lin and Peddada 2020, 2024, Zhou *et al.* 2022). The estimation procedures of these bias correction factors are derived based on distribution assumptions of absolute abundance fold changes among all the taxa. This strategy is effective when the distribution assumptions are close to the truth. Otherwise, it will lead to high false discovery rates. A third group of methods normalizes counts with one or multiple reference taxa (Sohn *et al.* 2015, Brill *et al.* 2022, Yang and Chen 2022). The reference taxa are assumed to be not DA. This idea is simple and effective (Morton *et al.* 2019). However, existing reference taxa selection procedures rely on calculating log count ratios between taxa pairs, which is computationally expensive and inaccurate, given the excessive zero values.

We have developed a new DAA method called “Analysis of Microbiome Differential Abundance by Pooling Tobit Models” (ADAPT). ADAPT has two innovations. First, we treat the zero counts as left-censored at the detection limit of the sequencing instrument. A censored observation unifies different zero mechanisms and accurately reflects the information contained in the observation. A similar idea was proposed for comparing relative abundances using Cox proportional hazards models (Chan and Li 2024). As far as we are concerned, the censoring perspective has not been investigated for absolute abundance comparison. Second, we introduce a novel way of finding non-differentially abundant (non-DA) reference taxa and using reference taxa to identify DA ones. Under the common assumption that DA taxa are the minority (Yang and Chen 2022), we provide solid theoretical justification that selecting reference taxa is feasible based on the taxa’s relative abundances. We implement these two ideas by adopting the Tobit model (Tobin 1958) from econometrics and survival analysis. We generate synthetic microbiome count data from independent simulation frameworks and show that ADAPT has better control of false discovery rates and higher detection power than competitor methods. We also demonstrate our method on the saliva and plaque samples of infants in the COHRA2 (Neiswanger *et al.* 2015) study to reveal DA taxa between kids who developed early childhood dental caries (ECC) and those who did not.

## 2 Materials and methods

We first illustrate the overall workflow of ADAPT with a toy example in Section 2.1. We then lay out the mathematical properties of relative abundance in Section 2.2. These mathematical properties of relative abundance pave the way for the detailed procedures of ADAPT in Section 2.3.

### 2.1 Toy example

There are seven taxa in the toy example (Fig. 1), and three are DA between two ecosystems. We aim to identify the three DA taxa based on the observed counts in the two metagenomics samples. We denote the counts of undetected taxa 6 and 7 in sample one as left-censored at one (the detection limit, which is assumed to be known). Therefore, their relative abundances are left-censored at 1/21. Similarly, we denote the count of taxon 4 in sample two as left-censored at one and its relative abundance to be left-censored at 1/35. We calculate the relative abundance fold change between two samples for all the taxa. According to the assumption that a minority of taxa are DA, we can be confident that taxa with median relative abundance fold changes are not DA (see Section 2.2 and Section S1 of Supplementary data for theoretical justifications). We choose these taxa as reference taxa. The reference taxa are taxa 2, 3, and 5 in this example. We normalize the individual taxa counts with the sum of reference taxa. By comparing the normalized counts between the two samples, we can correctly identify taxa 1, 4, and 7 as DA without including false positives.

When analyzing real-life microbiome data with more samples and taxa, we introduce Tobit models for modeling potentially left-censored relative abundances and normalized counts. We pool the effect size estimates and the hypothesis test *P*-values of Tobit models to find reference taxa and identify DA taxa. The detailed procedures of ADAPT are described in Section 2.3.

### 2.2 Mathematical properties of relative abundance

The intuition of ADAPT is supported by mathematical properties of relative abundance. We first present four propositions about relative abundance, which set the stage for deriving ADAPT analysis procedures. The proofs for these four propositions are in Section S1 of Supplementary data.

Let $A_{j}^{\left(g\right)}$ and $R_{j}^{\left(g\right)}$ represent the absolute abundance and relative abundance of taxon *j* (j=1,2,…,P) in condition *g* (g=1,2). The goal of DAA is to decide if $A_{j}^{\left(2\right)}$ is different from $A_{j}^{\left(1\right)}$ for each taxon *j*. The absolute abundances cannot be directly measured, but the relative abundances can be estimated from the observed sequencing counts. According to the definition, $R_{j}^{\left(g\right)}=A_{j}^{\left(g\right)}/\sum_{j^{\prime }=1}^{P}A_{j^{\prime }}^{\left(g\right)}$.

Proposition 1 (**Reference taxa**).

*Let* $T_{0}\subseteq T=\{1,2,\ldots ,P\}$  *be a set of non-DA taxa. Then, the relationship between the relative abundance fold changes and the absolute abundance fold changes of any taxon j (*j=1,2,…,P*) satisfies*
 

$$\frac{R_{j}^{\left(2\right)}/\sum_{k\in T_{0}}R_{k}^{\left(2\right)}}{R_{j}^{\left(1\right)}/\sum_{k\in T_{0}}R_{k}^{\left(1\right)}}=\frac{A_{j}^{\left(2\right)}}{A_{j}^{\left(1\right)}}\;\forall j\in \{1,2,\ldots ,P\}$$

We can calculate the absolute abundance fold change for any taxa from their relative abundances if we find a subset of non-DA taxa as reference taxa. The calculation involves the relative abundance ratio between the taxon of interest and the sum of reference taxa.

Proposition 2 (**Null Case**).

*If none of the taxa* T={1,2,…,P}  *are DA, then the relative abundances of all the taxa remain the same across conditions. On the other hand, if the relative abundances of all the taxa remain the same between conditions, then the absolute abundance fold changes of all the taxa are the same. Namely*,

$$\begin{array}{cc} A_{j}^{\left(2\right)}=A_{j}^{\left(1\right)}\;\forall j\in \{1,2,\ldots ,P\} & \Rightarrow R_{j}^{\left(2\right)}=R_{j}^{\left(1\right)}\;\forall j\in \{1,2,\ldots ,P\}\\ R_{j}^{\left(2\right)}=R_{j}^{\left(1\right)}\;\forall j\in \{1,2,\ldots ,P\} & \Rightarrow \frac{A_{1}^{\left(2\right)}}{A_{1}^{\left(1\right)}}=\frac{A_{2}^{\left(2\right)}}{A_{2}^{\left(1\right)}}=\cdots =\frac{A_{P}^{\left(2\right)}}{A_{P}^{\left(1\right)}} \end{array}$$

Suppose the relative abundances of all the taxa remain the same between conditions. In that case, all taxa’s absolute abundance fold changes may equal a constant other than one. However, most taxa are assumed to be non-DA (Kumar *et al.* 2018, Yang and Chen 2022). Based on this assumption, we can decide that there are no DA taxa if no relative abundances change between conditions for any taxa.

Proposition 3 (**Order preservation for abundance fold changes**).

*Between any two taxa j and k (*1≤j<k≤P*), their order of relative abundance fold changes is the same as their order of absolute abundance fold changes. Namely*
 

$$\frac{R_{j}^{\left(2\right)}}{R_{j}^{\left(1\right)}}\leq \frac{R_{k}^{\left(2\right)}}{R_{k}^{\left(1\right)}}\;\Leftrightarrow \;\frac{A_{j}^{\left(2\right)}}{A_{j}^{\left(1\right)}}\leq \frac{A_{k}^{\left(2\right)}}{A_{k}^{\left(1\right)}}$$

Relative abundance fold change is different from absolute abundance fold change. Still, relative abundance fold change is ranked the same among all taxa as absolute abundance fold change.

Proposition 4 (**Relative abundance fold change of non-DA taxa**).

*Under the assumption that a minority of taxa are DA, the relative abundance fold change of a non-DA taxon j (*j=1,2,…,P*) equals the median of the relative abundance fold changes of all taxa. Namely*,

$$R_{j}^{\left(2\right)}/R_{j}^{\left(1\right)}=\text{Median}{\left\{R_{j^{\prime }}^{\left(2\right)}/R_{j^{\prime }}^{\left(1\right)}\right\}}_{j^{\prime }=1,2,\ldots ,P}\Leftrightarrow A_{j}^{\left(2\right)}=A_{j}^{\left(1\right)}$$

The relative abundances of non-DA taxa differ between two conditions when DA taxa exist. Nevertheless, we can rank the relative abundance fold changes and select taxa with median fold changes as reference taxa.

### 2.3 ADAPT procedures

Proposition 1 indicates that a reference set consisting of non-DA taxa is sufficient to perform inference on the absolute abundances based solely on relative abundance. Then, Proposition 4 suggests that the median relative abundance fold change can be used as a criterion for constructing a reference set. However, metagenomics data are noisy in practice, which implies that generally only a single taxon will have a fold change equal to the median. Since a large reference set is desirable to reduce both the variance and the chance of choosing a DA taxon as reference, we must relax the equality criterion to a more lenient inclusion procedure that still selects only non-DA taxa. In particular, by our assumption, the reference set could be ideally composed of up to half the taxa in the study. Additionally, Proposition 2 provides a way to decide if none of the taxa are DA by investigating only the relative abundances.

ADAPT integrates these observations into three main procedures, leading to a complete DAA method. The first step estimates the relative abundance fold changes of all the taxa with Tobit models and decides if any DA taxa exist, in accordance with Proposition 2. Provided the first step confirms the existence of at least one DA taxa, the second step selects a subset of presumed non-DA taxa as reference taxa, based on the intuition from Proposition 4. Finally, the third step identifies DA taxa by fitting Tobit models to the log count ratios between each taxon and the reference taxa. We refer the reader to Supplementary Fig. S1 for a flowchart of the full methodology.

#### 2.3.1 Relative abundance fold change estimation with Tobit models

The metagenomics count table *Y* has *N* samples and *P* taxa. The count of taxon *j* (j=1,2,…,P) in sample *i* (i=1,2,…,N) is denoted as $y_{ij}$. Each sample *i* has its vector of covariates $x_{i}$ including the intercept. The main variable of interest $x_{i1}$ is binary for DAA between two conditions. If $y_{ij}$ is zero, we represent it as being left-censored at a positive value *d*


$$y_{ij}^{*}=\{\begin{array}{cc} d & \text{if}\;y_{ij}=0\\ y_{ij} & \text{if}\;y_{ij}>0 \end{array}\;\delta_{ij}=\left\{ \begin{array}{c} 0\;\text{if}\;y_{ij}=0\\ 1\;\;\text{if}\;y_{ij}>0 \end{array}\right.$$

The default value of *d* is one because metagenomics sequencing counts are integers and the detection limit of the sequencing instrument is one. The relative abundance of taxon *j* in sample *i* is denoted $z_{ij}=y_{ij}^{*}/\sum_{j^{\prime }=1}^{P}y_{ij^{\prime }}$. We fit a Tobit model (Tobin 1958) to the log relative abundances ${\left\{\;\text{log}\;z_{ij}\right\}}_{i=1,2,\ldots ,N}$ of each taxon j∈{1,2,…,P} by calculating the maximum likelihood estimate of


$$L(\beta_{j},\sigma_{j})=\prod_{i=1}^{N}{\left[\phi \left(\frac{\;\text{log}\;z_{ij}-x_{i}^{⊤}\beta_{j}}{\sigma_{j}}\right)\right]}^{\delta_{ij}}{\left[\Phi \left(\frac{\;\text{log}\;z_{ij}-x_{i}^{⊤}\beta_{j}}{\sigma_{j}}\right)\right]}^{1-\delta_{ij}}$$

where ϕ(·) and Φ(·) represent the probability density function and cumulative distribution of the standard normal distribution. The effect sizes of all covariates are included in $\beta_{j}$. The scale parameter σ accounts for the variance of log relative abundances. To guarantee the numerical stability of model fitting for rare taxa, we estimate the MLE of the Firth penalized likelihood (Alam *et al.* 2022). Section S2 of Supplementary data describes the computational details.

We report the effect size estimate ${\hat{\beta }}_{j1}$ which represents the log relative abundance fold change of taxon *j* between conditions. We also carry out likelihood ratio testing of $H_{0}:\beta_{j1}=0$ against $H_{1}:\beta_{j1}\neq 0$ and report the *P*-value. We pool the *P*-values of all the Tobit models to find DA taxa in the following steps.

#### 2.3.2 Reference taxa selection

If there are no DA taxa, the *P*-values of hypothesis tests for relative abundance fold changes in the first step ${\left\{w_{j}\right\}}_{j=1,2,\ldots ,P}$ should display a uniform distribution according to Proposition 2. We fit a beta-uniform mixture (Pounds and Morris 2003)


w∼πU(0,1)+(1−π)Beta(α,1) 0<π≤1,0<α<1

and apply likelihood ratio tests for $H_{0}:\pi =1$ against $H_{1}:\pi <1$. If $H_{0}$ cannot be rejected, the distribution of ${\left\{w_{j}\right\}}_{j=1,2,\ldots ,P}$ follows a uniform or left-skewed distribution, indicating that there are no DA taxa.

If $H_{0}$ is rejected, the distribution of *P*-values is right-skewed and there are DA taxa. We must then search for a subset of reference taxa before identifying DA taxa. According to Propositions 3 and 4, a taxon *j* is likely non-DA if its relative abundance fold change estimate ${\hat{\beta }}_{j1}$ is close to $\text{median}{\left\{{\hat{\beta }}_{j^{\prime }1}\right\}}_{j^{\prime }=1,2,\ldots ,P}$. Therefore, we select a subset $T^{\prime }$ with half of all the taxa whose relative abundance fold change estimates are closest to the median.


$$\begin{array}{c} d_{j}=\left|{\hat{\beta }}_{j1}-\text{median}{\left\{{\hat{\beta }}_{j^{\prime }1}\right\}}_{j^{\prime }=1,2,\ldots ,P}\right|\\ T^{\prime }=\{k|d_{k}<\text{median}{\left\{d_{j}\right\}}_{j=1,2,\ldots ,P}\} \end{array}$$

We verify if there are any DA taxa in $T^{\prime }$ in a way similar to the first step. For each taxon $k\in T^{\prime }$, we fit Tobit models to the count proportion within this subset $z_{ik}^{\prime }=y_{ik}^{*}/\sum_{k^{\prime }\in T^{\prime }}y_{ik^{\prime }}$. We then check if the distribution of *P*-values from these Tobit models is still right-skewed. If the *P*-value distribution is uniform or left-skewed, $T^{\prime }$ is a qualified set of reference taxa. Otherwise, we repeat halving until we obtain a subset of non-DA taxa $T_{0}$ as reference taxa.

#### 2.3.3 Identification of DA taxa

Once we identify a subset of non-DA taxa as the reference set, we calculate the count ratio between each taxon *j* and the summed counts of the reference taxa $u_{ij}=y_{ij}^{*}/\sum_{j^{\prime }\in T_{0}}y_{ij^{\prime }}$ for all samples i=1,2,…,N. We fit a Tobit model to ${\left\{\;\text{log}\;u_{ij}\right\}}_{i=1,2,\ldots ,N}$ by calculating the maximum likelihood estimate of


$$L(\gamma_{j},\psi_{j})=\prod_{i=1}^{N}{\left[\phi \left(\frac{\;\text{log}\;u_{ij}-x_{i}^{⊤}\gamma_{j}}{\psi_{j}}\right)\right]}^{\delta_{ij}}{\left[\Phi \left(\frac{\;\text{log}\;u_{ij}-x_{i}^{⊤}\gamma_{j}}{\psi_{j}}\right)\right]}^{1-\delta_{ij}}$$

The effect size estimate ${\hat{\gamma }}_{j1}$ represents the log fold change of absolute abundance for taxon *j* according to Proposition 1. The hypothesis test $H_{0}:\gamma_{j1}=0$ against $H_{1}:\gamma_{j1}\neq 0$ indicates whether taxon *j* is DA. We apply multiple testing corrections to all the *P*-values to control false discovery rates and call a taxon DA if its adjusted *P*-value is below a certain level (e.g., .05).

## 3 Results

### 3.1 Simulation studies

We evaluate the false positive rate control, false discovery rate control, and statistical power of ADAPT with synthetic data. We generate synthetic data using the simulation framework SparseDOSSA (Ma *et al.* 2021). SparseDOSSA draws absolute abundances of taxa from zero-inflated log-normal distributions and draws library sizes of all samples from log-normal distributions. The metagenomics sequencing counts are drawn from multinomial distributions based on the simulated library sizes and absolute abundances. The parameters for the simulations are estimated from 16S rRNA sequencing of stool samples in the Human Microbiome Project (Schiffer *et al.* 2019). We prepare accompanying metadata with a binary covariate and a continuous covariate. The binary covariate represents two contrasting conditions. The zero inflation probabilities and the means of log-normal distribution correlate with this binary covariate for DA taxa. The continuous covariate is a potentially confounding variable. It may be correlated with the metadata’s binary variable of interest and some taxa’s absolute abundances. The details of the simulation setup are described in Section S3 of Supplementary data.

We compare the performance of ADAPT with eight other DAA methods. The competitors are ALDEx2 (Fernandes *et al.* 2014), MaAsLin2 (Mallick *et al.* 2021), metagenomeSeq (Paulson *et al.* 2013), ANCOM (Mandal *et al.* 2015), ZicoSeq (Yang and Chen 2022), DACOMP (Brill *et al.* 2022), ANCOMBC (Lin and Peddada 2020), and LinDA (Zhou *et al.* 2022). CAMP (Chan and Li 2024), the differential relative abundance method that treats zeros as censored observations, is also included in the comparison. These competitors represent a variety of solutions to the excessive zeros and compositionality of metagenomics data (Supplementary Table S1). The proportion of DA taxa, sample sizes, fold changes of absolute abundances, library sizes, and confounding covariates could impact the performance of DAA methods. Therefore, we prepare various scenarios to study their influence. When investigating the influence of one factor, all other factors are fixed. We prepare 500 replicates for each simulation setting and report the average of performance metrics.

**Figure 2.**
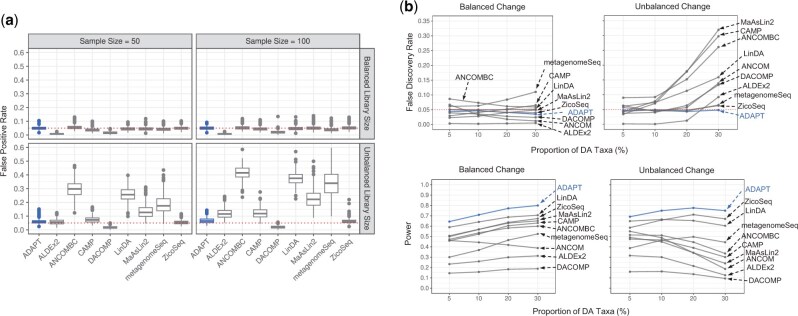
Simulation studies for comparing ADAPT with nine other DAA methods. We simulate synthetic metagenomics sequencing count data under two contrasting conditions using the SparseDOSSA framework. The number of samples is the same between the two conditions. We generate 500 replicates for each simulation setting and report the mean of performance metrics. **(a)** False positive rates (type I errors) of all methods except for ANCOM under simulation settings with no DA taxa. The total number of taxa is 500. The total sample size is 50 or 100. The average library size is the same (balanced) for two conditions at $10^{4}$ or different (unbalanced) between two conditions ($10^{4}$ for one condition and $10^{5}$ for the other). **(b)** False discovery rates and power under simulation settings with different proportions of DA taxa. The sample size is 100. The total number of taxa is 500. The proportion of DA taxa is 5%, 10%, 20%, or 30%. The average library size is $2\times 10^{4}$ for both conditions. The average absolute abundance fold change of DA taxa is 5. The directions of absolute abundance changes of DA taxa may be balanced or unbalanced.

We first evaluate the false positive rates (type I errors) of all the DAA methods when there are no DA taxa between the two conditions (Fig. 2a). Because ANCOM does not report raw *P*-values for DAA, it is excluded from this comparison. When the average library sizes are the same between the two conditions, all the DAA methods can control the FPRs at or below the nominal level of .05. When the library sizes are unbalanced, the proportion of sampling zeros and biological zeros differs between conditions. Competitors such as ANCOMBC and LinDA, which replace all the zeros with constant pseudo-counts, have the most inflated FPRs. The zero-inflated model by metagenomeSeq also struggles to decipher the zero mechanisms that are confounded by library sizes. DAA methods detect more DA taxa when the sample sizes are larger, which leads to more severely inflated FPRs for some of the competing methods. ADAPT can maintain FPRs around the nominal level regardless of the unbalanced average library sizes between conditions. After applying the Benjamini–Hochberg correction to the *P*-values, ADAPT can correctly decide that there are no DA taxa with over 95% accuracy if the library sizes are the same between the two conditions (Supplementary Fig. S2). If the library sizes are significantly different between conditions, the accuracy remains high at around 90%. When ADAPT accidentally reports the existence of DA taxa, it rarely identifies more than one DA taxon. This experiment shows that left censoring by ADAPT is more robust than ad hoc zero replacement strategies at handling excessive zeros in metagenomics data.

We then evaluate the false discovery rate control and power of all DAA methods when we experiment with different proportions of DA taxa (Fig. 2b). In simulation settings with balanced changes, most DAA methods can control FDR regardless of the proportion of DA taxa. The power of most DAA methods except for ANCOM increases as the proportion of DA taxa increases. Many competing DAA methods cannot control FDRs in simulation settings with unbalanced changes, especially when DA taxa proportions are high. This is because many normalization strategies by competing methods assume that the number of taxa whose absolute abundances are enriched is similar to those whose absolute abundances are depleted between two conditions. This assumption is violated when the changes of all DA taxa are in the same direction. ADAPT does not make assumptions about the distributions of absolute abundance fold changes and consistently selects non-DA taxa as reference taxa. The robust selection scheme of reference taxa guarantees false discovery rate control. ADAPT also has the highest average detection power.

To confirm the consistency of the observed trend, we repeat the two simulations with another simulation framework MIDASim (He *et al.* 2024). MIDASim introduces correlations between taxa abundances and assumes truncated generalized gamma distributions for the relative abundances of individual taxa (more details in Section S3.3 of Supplementary data). ADAPT maintains excellent control of FPR when there are no DA taxa (Supplementary Figs S3 and S4). Its FDR stays near the nominal level of 0.05 regardless of the proportion of DA taxa (Supplementary Fig. S5). The power of ADAPT is higher than almost all the other DAA methods, indicating its competitiveness. The closest competitor is ZicoSeq. ZicoSeq has higher power than ADAPT when recovering DA taxa but suffers from inflated false positive rates when analyzing data with no DA taxa and unbalanced library sizes.

Other factors affect the FDR control and power besides the proportion of DA taxa. The detection power of all DAA methods decreases drastically as the sample sizes decrease (Supplementary Fig. S6). Several competing methods, such as metagenomeSeq and ANCOMBC, have inflated FDRs when the sample size is as small as 50. The detection power of all methods increases as the average absolute abundance fold changes of DA taxa increase (Supplementary Fig. S7). Increasing the average library sizes of samples boosts the detection power (Supplementary Fig. S8). If the absolute abundances are affected by confounding covariates, DAA methods must adjust for the confounders to control FDRs (Supplementary Fig. S9). ADAPT has the most consistent control of FDRs and the highest average power across all the simulation scenarios. The computation times of all the DAA methods are mainly determined by the total number of taxa. ADAPT has the best computational efficiency among all the competitors (Supplementary Fig. S10). It only takes ADAPT 0.176 seconds to analyze a count table with 1000 taxa and 100 samples. ZicoSeq, which has the best balance of FDR control and power among all competitors, needs 80 seconds.

### 3.2 Real data analysis: ECC

Dental caries is the most common chronic disease for US children aged 5–17 (Ribeiro and Paster 2023). Supragingival microbial communities are associated with ECC (Xu *et al.* 2014). We can use DAA to identify microbiome taxa whose abundances differ between children who developed ECC by 5 years old and those who did not. We use 16S rRNA sequencing data from saliva samples and shotgun metagenomics sequencing (WGS) data from plaque samples of the Center for Oral Health Research in Appalachia 2 (COHRA2) cohort (Blostein *et al.* 2022). There are 161 saliva samples collected at 12 months old. None of the 161 children had dental caries during sample collection. Among these children, 84 later developed ECC, and 77 did not. There are 30 plaque samples collected between 36 and 60 months old. Half of the 30 samples were collected from children with dental caries, and the other half were caries-free. The plaque samples of the cases were collected at the onset of ECC. We remove taxa with prevalence lower than 5%, leading to 155 amplicon sequence variants (ASVs) in the count table of the saliva samples and 590 taxa in the count table of the plaque samples. We apply all the nine methods compared in the simulation studies and carry out DAA for the saliva samples and plaque samples separately. We apply the Benjamini–Hochberg correction to the raw *P*-values for all the DAA methods and use .05 as the cutoff level for DA taxa identification.

**Figure 3.**
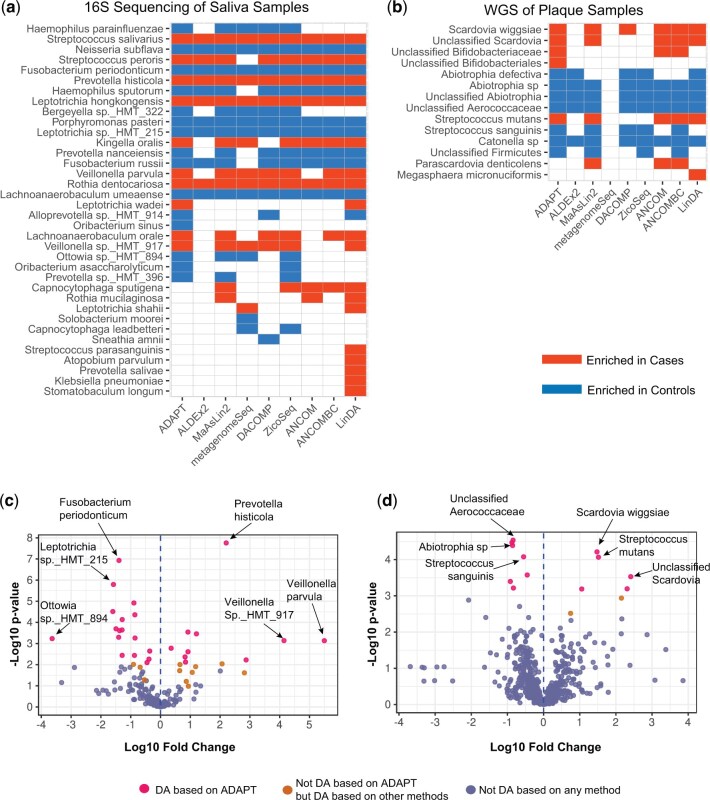
Microbiome DAA between children who developed ECC and those who did not. **(a)** Thirty-eight out of 155 total ASVs in the saliva samples collected at 12 months old are DA based on at least one method. ADAPT detects 27 DA ASVs. **(b)** Fourteen out of 590 taxa in the plaque samples collected between 36 and 60 months old are DA based on at least one method. ADAPT detects 12 DA taxa. **(c)** Volcano plot for DAA of saliva samples. **(d)** Volcano plot for DAA of plaque samples.

Among the 155 ASVs in the saliva samples, 38 are identified as DA by at least one DAA method (Fig. 3a, Supplementary Table S2). ADAPT identifies 27 DA ASVs. Several ASVs discovered by ADAPT were mentioned in multiple previous studies according to a recent review (Bhaumik *et al.* 2021), including *Haemophilus parainfluenzae*, *Fusobacterium periodonticum*, *Prevotella histicola*, *Veillonella parvula*, *Lachnoanaerobaculum umeaense*, and *Porphyromonas pasteri*. Among these six species, *H. parainfluenzae*, *F. periodonticum*, *L. umeaense*, and *P. pasteri* are enriched in children free of dental caries. *P. histicola* and *V. parvula* are enriched in children who later developed dental caries. These trends align with findings in previous literature as well.

Among the 590 taxa in the plaque samples, 14 are identified as DA by at least one DAA method (Fig. 3b, Supplementary Table S3). ADAPT identifies 12 DA taxa. The discoveries of ADAPT include *Scardovia wiggsiae*, *Streptococcus mutans*, and *Streptococcus sanguinis*, which were mentioned in previous reviews (Bhaumik *et al.* 2021). According to ADAPT, *S. sanguinis* is enriched in controls. *S. mutans* and *S. wiggsiae* are enriched in cases. These trends agree with previous findings as well. The DA taxa in the plaque samples collected after 36 months old differ from the DA taxa in the pre-incident saliva samples collected at 12 months old. This phenomenon echoes the idea that microbiome species associated with dental caries vary with age (Bhaumik *et al.* 2021, Blostein *et al.* 2022).

ADAPT estimates the absolute abundance fold changes besides identifying DA taxa. Most taxa’s estimated log10 fold changes are between −2 and 2 for both the saliva and the plaque samples (Fig. 3c and d). For some rare taxa that only exist in samples from one condition, the absolute values of their estimated effect sizes are much larger than the others. For example, *Veillonella parvula* has the largest estimated log10 fold change of 5.50 among all the ASVs in the saliva samples. This species was detected in 10 of the 161 saliva samples, and all these 10 samples were from children who eventually developed dental caries. The count proportions of *Veillonella parvula* in these 10 samples range from $6\times 10^{-4}$ to 0.06. Still, complete separation does not necessarily indicate DA taxa. For example, *Fusobacterium naviforme* is detected only in three of the 30 plaque samples, all from children without dental caries. The estimated log10 fold change is −3.687, the lowest among all the taxa. However, the corresponding *P*-value is .09, so *Fusobacterium naviforme* is not considered DA. The count proportions of *Fusobacterium naviforme* among the three samples are only $5.8\times 10^{-6}$, $1.0\times 10^{-6}$, and $3.1\times 10^{-7}$. The computational heuristics in ADAPT prevent infinite log abundance fold change estimates and enable valid statistical tests when complete separation occurs (Section S2 of Supplementary data). Analyses of real-life data show that ADAPT is robust and numerically stable for evaluating differential abundance patterns of rare taxa.

## 4 Discussion

The excessive zeros in metagenomics data come from multiple sources, and it is challenging to classify and preprocess them for DAA (Kaul *et al.* 2017, Silverman *et al.* 2020, Jiang *et al.* 2022). Left censoring can circumvent any controversial zero preprocessing steps. ADAPT is the first method to demonstrate the ingenuity of censoring when comparing absolute abundances. The simulation studies and real-data analyses prove that censoring can control false discovery rates and maintain competitive detection power. We choose to censor all the observed zero counts at one (the smallest nonzero value) for all the simulations and real-data analyses. This proxy is a natural choice, given that metagenomics counts are discrete. Nevertheless, other choices are worth exploring. For example, we may censor the zeros at the smallest positive value in the count table if the smallest positive value does not equal one. We may also customize sample-specific proxies, given the library size and the smallest positive count in each sample. The prevalence of each taxon is another factor worth considering.

Reference taxa selection in ADAPT aims to find a subset of non-DA taxa. In reality, the reference taxa might contain a small number of DA ones. The criterion for a qualified reference taxa set is that the hypothesis test *P*-values form a uniform or left-skewed distribution. Because the decision is based on the distribution of *P*-values instead of thresholds for individual *P*-values, a handful of DA taxa is expected to remain in the reference taxa set. Simulations confirm that contamination within the reference taxa is much lower than the proportion of DA taxa among all the taxa (Supplementary Fig. S11). The most ideal choice of reference taxa set is the one with all the non-DA taxa. This choice is not feasible for real data but is available in simulations. In the simulations, the DA taxa identified by the oracle model with all the non-DA taxa as reference highly overlap with the ones identified by ADAPT (Supplementary Fig. S12). There is no significant difference in false discovery rates between the outcomes based on two different reference choices (Supplementary Fig. S13). The difference in power is also small except for simulations with unbalanced changes and high proportions of DA taxa (Supplementary Fig. S14). These closer inspections illustrate that the reference taxa selection scheme of ADAPT is robust.

The Tobit model is similar to the accelerated failure time model in survival analysis, except that it models left-censored rather than right-censored data. It is a parametric censored quantile regression. The simulation studies and real data analyses consider microbiome differential abundance between two conditions, but ADAPT can also handle continuous conditions and adjust for multiple covariates. In future work, we will accommodate multigroup comparisons and include random effects for longitudinal study designs. Nonparametric censored quantile regressions (Portnoy 2003, Peng and Huang 2008) are viable alternatives to the Tobit model. They could be more robust than the Tobit model when the distribution of log count ratios departs from a normal distribution or when sample sizes are small. The successful implementation of ADAPT offers new perspectives on handling excessive zeros and compositional data for microbiome DAA. It is a valuable addition to the field of ecological data analysis.

## Supplementary Material

btae661_Supplementary_Data

## Data Availability

The 16S rRNA sequencing data and shotgun metagenomics sequencing data of the COHRA2 study (Blostein *et al.* 2022) are available under project number PRJNA752888 (https://www.ncbi.nlm.nih.gov/bioproject/PRJNA752888). The metadata can be requested from the dbGaP database under study accession number phs001591.v1.p1. The preprocessed count tables and de-identified metadata for the metagenomics data in the ECC study are available through the ADAPT R package (https://github.com/mkbwang/ADAPT).
