# Supplementary material for: ADAPT: Analysis of Microbiome Differential Abundance by Pooling Tobit Models
Source: Bioinformatics. 2024 Nov 7;40(11):btae661. doi: 10.1093/bioinformatics/btae661 (PMC11959182; doi:10.1093/bioinformatics/btae661)
Supplement: btae661_Supplementary_Data [file btae661_supplementary_data.zip › ADAPT_Supplementary_Notes_Figures.pdf]

# Supplementary Notes and Figures for "ADAPT: Analysis of Microbiome Differential Abundance by Pooling Tobit Models"

## S1 Proofs for Propositions

This section contains the proofs for the four propositions about relative abundance.

*Proof for Proposition 1.* We know that
$R_j^{(g)} = A_j^{(g)} / \sum_{j'=1}^{P} A_{j'}^{(g)}$ and $\sum_{k \in \mathcal{T}_0} R_k^{(g)} = \sum_{k \in \mathcal{T}_0} A_k^{(g)} / \sum_{j'=1}^{P} A_{j'}^{(g)}$, therefore

$$\frac{R_j^{(g)}}{\sum_{k \in \mathcal{T}_0} R_k^{(g)}} = \frac{A_j^{(g)}}{\sum_{k \in \mathcal{T}_0} A_k^{(g)}}$$

Furthermore

$$\frac{R_j^{(2)} / \sum_{k \in \mathcal{T}_0} R_k^{(2)}}{R_j^{(1)} / \sum_{k \in \mathcal{T}_0} R_k^{(1)}} = \frac{A_j^{(2)} / \sum_{k \in \mathcal{T}_0} A_k^{(2)}}{A_j^{(1)} / \sum_{k \in \mathcal{T}_0} A_k^{(1)}}$$

Because all the taxa in $\mathcal{T}_0$ are non-DA, $\sum_{k \in \mathcal{T}_0} A_k^{(2)} = \sum_{k \in \mathcal{T}_0} A_k^{(1)}$, therefore

$$\frac{R_j^{(2)} / \sum_{k \in \mathcal{T}_0} R_k^{(2)}}{R_j^{(1)} / \sum_{k \in \mathcal{T}_0} R_k^{(1)}} = \frac{A_j^{(2)}}{A_j^{(1)}}$$

□

*Proof for Proposition 2.* If $A_j^{(2)} = A_j^{(1)} \quad \forall j \in \{1, 2, \cdots, P\}$, then the total microbial loads are the same between two conditions as well, namely $\sum_{j'=1}^{P} A_{j'}^{(2)} = \sum_{j'=1}^{P} A_{j'}^{(1)}$. We can deduce that

$$R_j^{(2)} = \frac{A_j^{(2)}}{\sum_{j'=1}^{P} A_{j'}^{(2)}} = \frac{A_j^{(1)}}{\sum_{j'=1}^{P} A_{j'}^{(1)}} = R_j^{(1)}$$

On the other hand, if we know that the relative abundance $R_j^{(2)} = R_j^{(1)} \quad \forall j \in \{1, 2, \cdots, P\}$, we can get

$$\frac{A_j^{(2)}}{\sum_{j'=1}^{P} A_{j'}^{(2)}} = \frac{A_j^{(1)}}{\sum_{j'=1}^{P} A_{j'}^{(1)}} \quad \Rightarrow \quad \frac{A_j^{(2)}}{A_j^{(1)}} = \frac{\sum_{j'=1}^{P} A_{j'}^{(2)}}{\sum_{j'=1}^{P} A_{j'}^{(1)}} \quad \forall j \in \{1, 2, \cdots, P\}$$

This indicates that $\frac{A_1^{(2)}}{A_1^{(1)}} = \frac{A_2^{(2)}}{A_2^{(1)}} = \cdots = \frac{A_P^{(2)}}{A_P^{(1)}}$ □

*Proof for Proposition 3.* According to the definition of relative abundance,

$$\frac{R_j^{(2)}}{R_j^{(1)}} = \frac{A_j^{(2)} / \sum_{j'=1}^{P} A_{j'}^{(2)}}{A_j^{(1)} / \sum_{j'=1}^{P} A_{j'}^{(1)}} \qquad \frac{R_k^{(2)}}{R_k^{(1)}} = \frac{A_k^{(2)} / \sum_{j'=1}^{P} A_{j'}^{(2)}}{A_k^{(1)} / \sum_{j'=1}^{P} A_{j'}^{(1)}}$$

Therefore

$$\frac{R_j^{(2)}}{R_j^{(1)}} < \frac{R_k^{(2)}}{R_k^{(1)}} \quad \Leftrightarrow \quad \frac{A_j^{(2)} / \sum_{j'=1}^{P} A_{j'}^{(2)}}{A_j^{(1)} / \sum_{j'=1}^{P} A_{j'}^{(1)}} < \frac{A_k^{(2)} / \sum_{j'=1}^{P} A_{j'}^{(2)}}{A_k^{(1)} / \sum_{j'=1}^{P} A_{j'}^{(1)}} \quad \Leftrightarrow \quad \frac{A_j^{(2)}}{A_j^{(1)}} < \frac{A_k^{(2)}}{A_k^{(1)}}$$

□

*Proof for Proposition 4.* Because fewer than half of all the taxa are differentially abundant, we can be certain that the median absolute abundance fold change is equal to one, namely Median$\{A_{j'}^{(2)}/A_{j'}^{(1)}\}_{j'=1,2,\cdots,P} = 1$. Based on Proposition 3,

$$R_j^{(2)}/R_j^{(1)} = \text{Median}\{R_{j'}^{(2)}/R_{j'}^{(1)}\}_{j'=1,2,\cdots,P} \quad \Leftrightarrow \quad A_j^{(2)}/A_j^{(1)} = \text{Median}\{A_{j'}^{(2)}/A_{j'}^{(1)}\}_{j'=1,2,\cdots,P}$$

It immediately follows that

$$R_j^{(2)}/R_j^{(1)} = \text{Median}\{R_{j'}^{(2)}/R_{j'}^{(1)}\} \quad \Leftrightarrow \quad A_j^{(2)} = A_j^{(1)}$$

□

# S2 Computational Details of Tobit Model

The Tobit model [1] is the core of ADAPT. When analyzing rare taxa, many observations are left-censored, which may cause the standard estimation method for Tobit models to fail to converge. We adopt several computational heuristics to guarantee the Tobit model estimation's consistency and numerical stability.

## S2.1 Reparameterization

We use $\{Y_i, \delta_i\}_{i=1,2,\cdots,N}$ to denote the value and censorship indicator of $N$ samples. The censorship indicator $\delta_i$ equals zero if the observation is left censored and one otherwise. Each observation has its corresponding vector of covariates $\boldsymbol{x}_i$. Suppose there are $P$ covariates, including the intercept. The log-likelihood of the Tobit model is

$$\ell = \sum_{i=1}^{N} \delta_i \log \phi \left( \frac{Y_i - \boldsymbol{x}_i \boldsymbol{\beta}}{\sigma} \right) + \sum_{i=1}^{N} (1 - \delta_i) \log \Phi \left( \frac{Y_i - \boldsymbol{x}_i \boldsymbol{\beta}}{\sigma} \right) \tag{1}$$

where $\phi(\cdot)$ and $\Phi(\cdot)$ represent the probability density and cumulative distribution of standard normal distribution. This parameterization of the Tobit model with $\boldsymbol{\beta} = \begin{bmatrix} \beta_0 & \beta_1 & \cdots & \beta_{P-1} \end{bmatrix}^\top$ as the effect sizes and $\boldsymbol{\sigma}$ as the scale may lead to multiple solutions during numerical optimization of maximum likelihood estimation [2]. This problem can be avoided if we choose to reparameterize $\boldsymbol{\beta}$ and $\sigma$ with $\boldsymbol{\rho} = \boldsymbol{\beta}/\sigma$ and $\omega = 1/\sigma$ [3]. This parameterization guarantees that the log-likelihood is globally concave and has only one maximum likelihood estimate. The log-likelihood of the Tobit model thus becomes

$$\ell = \sum_{i=1}^{N} \delta_i \log \phi \left( \omega Y_i - \boldsymbol{x}_i \boldsymbol{\rho} \right) + \sum_{i=1}^{N} (1 - \delta_i) \log \Phi \left( \omega Y_i - \boldsymbol{x}_i \boldsymbol{\rho} \right) \tag{2}$$

The hypothesis test for one effect size in $\boldsymbol{\beta}$ such as $H_0 : \beta_1 = 0$ against $H_1 : \beta_1 \neq 0$ is equivalent to $H_0 : \rho_1 = 0$ against $H_1 : \rho_1 \neq 0$. The MLE of $\boldsymbol{\beta}$ is $\hat{\boldsymbol{\beta}} = \hat{\boldsymbol{\rho}}/\hat{\omega}$.

## S2.2 Fisher's Score and Information for log likelihood

We introduce the notation $Z_i = \omega Y_i - \boldsymbol{x}_i^\top \boldsymbol{\rho}$ to simplify the log-likelihood in formula 2 to

$$\ell = \sum_{i=1}^{N} \delta_i \log \phi \left( Z_i \right) + \sum_{i=1}^{N} (1 - \delta_i) \log \Phi \left( Z_i \right) \tag{3}$$

The partial derivatives of $Z_i$ over $\omega$ and $\boldsymbol{\rho}$ are

$$\partial Z_i/\partial\omega = Y_i \qquad \partial Z_i/\partial\boldsymbol{\rho} = -\boldsymbol{x}_i \tag{4}$$

The Fisher's score and information of log-likelihood contains partial derivatives of $\ell$ over $Z_i$

$$\frac{\partial\ell}{\partial Z_i} = -\delta_i Z_i + \frac{1-\delta_i}{\sqrt{2\pi}}\cdot\Phi^{-1}(Z_i)\cdot\exp(-\frac{1}{2}Z_i^2) \tag{5}$$

$$\frac{\partial^2\ell}{\partial Z_i^2} = -\delta_i - \frac{1-\delta_i}{2\pi}\cdot\Phi^{-2}(Z_i)\cdot\exp(-Z_i^2) - \frac{1-\delta_i}{\sqrt{2\pi}}\cdot Z_i\cdot\Phi^{-1}(Z_i)\cdot\exp(-\frac{1}{2}Z_i^2) \tag{6}$$

$$\begin{aligned}\frac{\partial^3\ell}{\partial Z_i^3} = \frac{1-\delta_i}{\sqrt{2}\cdot\pi^{3/2}}\cdot\Phi^{-3}(Z_i)\cdot\exp(-\frac{3}{2}Z_i^2) + \frac{3(1-\delta_i)}{2\pi}Z_i\cdot\Phi^{-2}(Z_i)\cdot\exp(-Z_i^2) - \\ \frac{1-\delta_i}{\sqrt{2\pi}}\cdot\Phi^{-1}(Z_i)\cdot\exp(-\frac{1}{2}Z_i^2) + \frac{1-\delta_i}{\sqrt{2\pi}}\cdot Z_i^2\cdot\Phi^{-1}(Z_i)\cdot\exp(-\frac{1}{2}Z_i^2)\end{aligned} \tag{7}$$

Fisher's score is the first derivative of log-likelihood $\ell$ over each parameter of $\boldsymbol{\rho}$ and $\omega$. Denote the score vector in the log likelihood as $\boldsymbol{U} = \left[\boldsymbol{U}_{\boldsymbol{\rho}}^\top \ U_\omega\right]^\top$. The first-order partial derivatives are

$$\boldsymbol{U}_{\boldsymbol{\rho}} = \frac{\partial\ell}{\partial\boldsymbol{\rho}} = \sum_{i=1}^{N}\frac{\partial\ell}{\partial Z_i}\cdot\frac{\partial Z_i}{\partial\boldsymbol{\rho}} = -\sum_{i=1}^{N}\frac{\partial\ell}{\partial Z_i}\boldsymbol{x}_i \tag{8}$$

$$U_\omega = \sum_{i=1}^{N}\frac{\delta_i}{\omega} + \sum_{i=1}^{N}\frac{\partial\ell}{\partial Z_i}\cdot\frac{\partial Z_i}{\partial\omega} = \sum_{i=1}^{N}\frac{\delta_i}{\omega} + \sum_{i=1}^{N}\frac{\partial\ell}{\partial Z_i}\cdot Y_i \tag{9}$$

The information matrix $\boldsymbol{I}$ equals the negative hessian matrix $\boldsymbol{H}$. The Hessian matrix $\boldsymbol{H}$ represents the second order partial derivatives of log-likelihood $\ell$ over all the parameters in $\boldsymbol{\rho}$ and $\omega$

$$\boldsymbol{I} = -\boldsymbol{H} = \begin{bmatrix} -\frac{\partial^2\ell}{\partial\boldsymbol{\rho}\partial\boldsymbol{\rho}^\top} & -\frac{\partial^2\ell}{\partial\boldsymbol{\rho}\partial\omega} \\ -\frac{\partial^2\ell}{\partial\omega\partial\boldsymbol{\rho}^\top} & -\frac{\partial^2\ell}{\partial\omega^2}\end{bmatrix} \tag{10}$$

The four components in $\boldsymbol{I}$ are

$$\boldsymbol{I}_{\boldsymbol{\rho}\boldsymbol{\rho}} = -\frac{\partial^2\ell}{\partial\boldsymbol{\rho}\partial\boldsymbol{\rho}^\top} = -\sum_{i=1}^{N}\frac{\partial^2\ell}{\partial Z_i^2}\cdot\boldsymbol{x}_i\boldsymbol{x}_i^\top \tag{11}$$

$$\boldsymbol{I}_{\omega\boldsymbol{\rho}}^\top = \boldsymbol{I}_{\boldsymbol{\rho}\omega} = -\frac{\partial^2\ell}{\partial\boldsymbol{\rho}\partial\omega} = \sum_{i=1}^{N}\frac{\partial^2\ell}{\partial Z_i^2}\cdot Y_i\boldsymbol{x}_i \tag{12}$$

$$I_{\omega\omega} = -\frac{\partial^2\ell}{\partial\omega^2} = -\sum_{i=1}^{N}\frac{\partial^2\ell}{\partial Z_i^2}\cdot Y_i^2 + \sum_{i=1}^{N}\frac{\delta_i}{\omega^2} \tag{13}$$

## S2.3 Firth Penalized likelihood

There are situations where a taxon is not observed in any samples from one specific condition. These situations are called complete separation. The complete separation will lead to monotone log-likelihood, and the Newton-Raphson algorithm for maximum likelihood estimation will not converge. To avoid these numerical issues, we add a Firth penalty [4, 5] to the log-likelihood and find the MLE of the penalized likelihood as our parameter estimate. The Firth penalty $\ell_F$ can be understood as Jeffrey's prior on the parameters. The MLE of the penalized likelihood $\ell^\star$ is almost the same as the MLE for $\ell$ except when the sample size is small and/or the existence of a taxon displays patterns of complete separation.

$$\ell^\star = \ell + \ell_F = \ell + \frac{1}{2}\log|\boldsymbol{I}| \tag{14}$$

The Fisher's score $\boldsymbol{U}^\star = \boldsymbol{U} + \boldsymbol{U}_F$. We have derived $\boldsymbol{U}$ in formula 8 and 9 of the previous section. The partial derivative of $\ell_F$ for $\rho_j$ $(j = 0, 1, 2, \cdots, P-1)$ and $\omega$ is

$$U_{F,\rho_q} = \frac{\partial \ell_F}{\partial \rho_q} = \frac{1}{2}\mathrm{Tr}\left(\boldsymbol{I}^{-1}\frac{\partial \boldsymbol{I}}{\partial \rho_q}\right) \tag{15}$$

$$U_{F,\omega} = \frac{\partial \ell_F}{\partial \omega} = \frac{1}{2}\mathrm{Tr}\left(\boldsymbol{I}^{-1}\frac{\partial \boldsymbol{I}}{\partial \omega}\right) \tag{16}$$

The partial derivative of information matrix $\boldsymbol{I}$ over $\rho_j$ and $\omega$ is derived based on the four components in formula 11, 12 and 13. In terms of $\partial \boldsymbol{I}/\partial \rho_q$,

$$\frac{\partial \boldsymbol{I}}{\partial \rho_q} = \begin{bmatrix} \frac{\partial \boldsymbol{I}_{\boldsymbol{\rho\rho}}}{\partial \rho_q} & \frac{\partial \boldsymbol{I}_{\boldsymbol{\rho}\omega}}{\partial \rho_q} \\ \frac{\partial \boldsymbol{I}^\top_{\omega\boldsymbol{\rho}}}{\partial \rho_q} & \frac{\partial \boldsymbol{I}_{\omega\omega}}{\partial \rho_q} \end{bmatrix} \tag{17}$$

$$\frac{\partial \boldsymbol{I}_{\boldsymbol{\rho\rho}}}{\partial \rho_q} = \sum_{i=1}^{N} \frac{\partial^3 \ell}{\partial Z_i^3} x_{iq} \cdot \boldsymbol{x}_i \boldsymbol{x}_i^\top \qquad \frac{\partial \boldsymbol{I}_{\boldsymbol{\rho}\omega}}{\partial \rho_q} = -\sum_{i=1}^{N} \frac{\partial^3 \ell}{\partial Z_i^3} x_{iq} Y_i \cdot \boldsymbol{x}_i \qquad \frac{\partial I_{\omega\omega}}{\partial \rho_q} = \sum_{i=1}^{N} \frac{\partial^3 \ell}{\partial Z_i^3} x_{iq} Y_i^2 \tag{18}$$

In terms of $\partial \boldsymbol{I}/\partial \omega$,

$$\frac{\partial \boldsymbol{I}}{\partial \omega} = \begin{bmatrix} \frac{\partial \boldsymbol{I}_{\boldsymbol{\rho\rho}}}{\partial \omega} & \frac{\partial \boldsymbol{I}_{\boldsymbol{\rho}\omega}}{\partial \omega} \\ \frac{\partial \boldsymbol{I}^\top_{\omega\boldsymbol{\rho}}}{\partial \omega} & \frac{\partial \boldsymbol{I}_{\omega\omega}}{\partial \omega} \end{bmatrix} \tag{19}$$

$$\frac{\partial \boldsymbol{I}_{\boldsymbol{\rho\rho}}}{\partial \omega} = -\sum_{i=1}^{N} \frac{\partial^3 \ell}{\partial Z_i^3} Y_i \cdot \boldsymbol{x}_i \boldsymbol{x}_i^\top \qquad \frac{\partial \boldsymbol{I}_{\boldsymbol{\rho}\omega}}{\partial \omega} = \sum_{i=1}^{N} \frac{\partial^3 \ell}{\partial Z_i^3} Y_i^2 \cdot \boldsymbol{x}_i \qquad \frac{\partial I_{\omega\omega}}{\partial \omega} = -\sum_{i=1}^{N} \frac{\partial^3 \ell}{\partial Z_i^3} Y_i^3 - \sum_{i=1}^{N} \frac{2\delta_i}{\omega^3} \tag{20}$$

### S2.4 BFGS Algorithm

We plan to use the Newton-Raphson algorithm to calculate the MLE of the penalized log-likelihood, but the analytic form of the second-order derivative is hard to derive. Therefore, we resort to the BFGS algorithm [6], a quasi-Newton method. We initialize all entries in $\boldsymbol{\rho}$ to be zero except for the intercept. The intercept $\rho_0$ has initial value $\bar{\boldsymbol{Y}}/\mathrm{SD}(\boldsymbol{Y})$. The inverse scale $\omega$ has initial value $1/\mathrm{SD}(\boldsymbol{Y})$. We set up the initial second-order derivative to be equal to the Hessian matrix of the standard log-likelihood of the Tobit model. The initialization approximates the second-order derivative of the penalized likelihood. The update of the Hessian matrix during each quasi-Newton iteration relies on the approximate Hessian in the previous step, the most recent step size of the parameters, and the most recent change of the first derivatives. The details of the BFGS algorithm are depicted in pseudocode 1

## S3 Simulation Framework

### S3.1 Metadata Generation

The metadata has two variables $\boldsymbol{X}$ and $\boldsymbol{C}$. Variable $X_i (i = 1, 2, \cdots, N)$ is a binary variable representing two contrasting conditions. It is the variable of interest. Variable $C_i$ is a potentially confounding continuous covariate. For each sample $i$, $X_i$ and $C_i$ are generated in the following way

$$\begin{aligned} h_i &\sim \mathcal{N}(0,1) \\ X_i &= \mathbb{I}(h_i > 0) \\ C_i &= \eta h_i + \sqrt{1-\eta^2}\mathcal{N}(0,1) \end{aligned}$$

where $\eta$ is the correlation parameter that controls the severity of confounding.

### S3.2 Simulation based on SparseDOSSA Framework

SparseDOSSA [7] generates the count table $\boldsymbol{Y}$ based on the metadata. The simulation scheme of SparseDOSSA first generates the absolute abundances of each taxon from a zero-inflated log-normal distribution.

**Algorithm 1** BFGS algorithm for estimating the Tobit model parameters

**Input** Response values of $N$ samples $\boldsymbol{Y}$ , Covariate matrix $\boldsymbol{X}$ with dimension $N \times P$, Taxon existence indicator vector $\boldsymbol{\delta}$ of length $N$, Convergence tolerance threshold $\epsilon$

$r \leftarrow 0$ ▷ Iteration counter
$\boldsymbol{\rho}^{(r)} \leftarrow \mathbf{0}$ ▷ Initialize $\boldsymbol{\rho}$
$\rho_0^{(r)} \leftarrow \bar{\boldsymbol{Y}}/\mathrm{SD}(\boldsymbol{Y})$ ▷ Initialize intercept
$\omega^{(r)} \leftarrow 1/\mathrm{SD}(\boldsymbol{Y})$ ▷ Initialize inverse scale
$\boldsymbol{\theta}^{(r)\top} \leftarrow \left[\boldsymbol{\rho}^{(r)\top} \ \omega^{(r)}\right]$ ▷ Concatenate all the parameters
Calculate $\ell^{\star(r)}$ based on formula 2, 10, 14 and their dependents with $\boldsymbol{\theta}^{(r)}$ ▷ Initial log likelihood
Calculate $\boldsymbol{U}^{\star(r)}$ based on formula 8, 9, 15, 16 and their dependents with $\boldsymbol{\theta}^{(r)}$ ▷ Initial Fisher score
Calculate $\boldsymbol{B}^{(r)}$ based on formula 10 and its dependents ▷ Initialize approximate hessian matrix
**do**
  $r \leftarrow r + 1$
  $\Delta\boldsymbol{\theta} \leftarrow -{\boldsymbol{B}^{(r-1)}}^{-1}\boldsymbol{U}^{(r-1)}$
  $\boldsymbol{\theta}^{(r)} \leftarrow \boldsymbol{\theta}^{(r-1)} + \Delta\boldsymbol{\theta}$ ▷ Quasi Newton Update
  Calculate $\ell^{\star(r)}$ based on formula 2, 10, 14 and their dependents with $\boldsymbol{\theta}^{(r)}$
  Calculate $\boldsymbol{U}^{\star(r)}$ based on formula 8, 9, 15, 16 and their dependents with $\boldsymbol{\theta}^{(r)}$
  $\Delta\boldsymbol{U} \leftarrow \boldsymbol{U}^{\star(r)} - \boldsymbol{U}^{\star(r-1)}$
  $\boldsymbol{B}^{(r)} \leftarrow \boldsymbol{B}^{(r-1)} + \frac{\Delta\boldsymbol{U}\Delta\boldsymbol{U}^\top}{\Delta\boldsymbol{U}^\top\Delta\boldsymbol{\theta}} - \frac{\boldsymbol{B}^{(r-1)}\Delta\boldsymbol{\theta}\Delta\boldsymbol{\theta}^\top\boldsymbol{B}^{(r-1)}}{\Delta\boldsymbol{\theta}^\top\boldsymbol{B}^{(r-1)}\Delta\boldsymbol{\theta}}$ ▷ BFGS update of the approximate hessian
  $\Delta\ell \leftarrow \ell^{\star(r)} - \ell^{\star(r-1)}$
**while** $\left|\Delta\ell/\ell^{\star(r-1)}\right| > \epsilon$

**Output** Estimated parameters $\boldsymbol{\rho}^{(r)}$, $\omega^{(r)}$ and penalized log likelihood $\ell^{\star(r)}$

For taxon $j(j = 1, 2, \cdots, P)$ in sample $i(i = 1, 2, \cdots, N)$,

$$m_{ij} \sim \mathcal{N}(0, 1)$$
$$A_{ij} = \begin{cases} 0 & \Phi(m_{ij}) < \theta_{ij} \\ \mathrm{Lognormal}(\mu_{ij}, \tau_j^2) & \Phi(m_{ij}) > \theta_{ij} \end{cases}$$

where $m_{ij}$ is a latent variable for deciding if $A_{ij} > 0$. The zero inflation probability $\theta_{ij}$ and the log-normal distribution parameter $\mu_{ij}$ depend on $X_i$ and $C_i$

$$\log\left(\frac{1-\theta_{ij}}{\theta_{ij}}\right) = \log\left(\frac{1-\theta_{0j}}{\theta_{0j}}\right) + X_i\gamma_{j1} + C_i\gamma_{j2}$$
$$\mu_{ij} = \mu_{0j} + X_i\gamma_{j1} + C_i\gamma_{j2}$$

Taxon $j$ is differentially abundant if $\gamma_{j1} \neq 0$. Its abundance is correlated with the potential confounder if $\gamma_{j2} \neq 0$. The parameters $\left\{(\mu_{0j}, \theta_{0j}, \tau_j^2)\right\}_{j=1,2,\cdots,P}$ are drawn from the pre-trained template in the SparseDOSSA package. The pre-trained template was calculated based on 16S rRNA sequencing of stool samples in the Human Microbiome Project [8, 9]. There are 332 sets of zero-inflated log-normal distribution parameters in the pre-trained template. DAA performance is indistinguishable among different methods if the simulated count table contains too many rare taxa. Therefore, we only draw (with replacement) from 54 sets of parameters whose zero inflation probabilities are below 50% to set up absolute abundance distributions for all taxa. The relative abundances can be derived based on the generated absolute abundances $R_{ij} = A_{ij}/\sum_{j'=1}^{P} A_{ij'}$. We draw the library sizes from another log-normal distribution and generate the taxon counts from a multinomial distribution

$$D_i \sim \mathrm{Lognormal}(\mu_D, \tau_D^2)$$
$$(y_{i1}, y_{i2}, \cdots, y_{iP}) \sim \mathrm{Multinom}(D_i, R_{i1}, R_{i2}, \cdots, R_{iP})$$

## S3.3 Simulation based on MIDASim Framework

MIDASim [10] introduces correlations in relative abundances between taxa based on real metagenomics data as a template. The correlation matrix $\boldsymbol{\rho}$ is calculated based on tetrachoric correlation [11] of presence-absence among each pair of taxa. For the marginal distribution of the relative abundance of each taxon, MIDASim uses an accelerated failure time model in survival analysis to model the inverse relative abundance. We denote the library size of sample $i$ as $M_i$ and define the inverse relative abundance for taxon $j$ in sample $i$ as $t_{ij}$

$$t_{ij} = \begin{cases} \frac{1}{R_{ij}} & R_{ij} > 0 \\ M_i & R_{ij} = 0 \end{cases}$$

Under the generalized gamma distribution assumption, for taxon $j$, $\log(t) = -\mu_j + \sigma_j v$ where $x = \exp(v)$ follows Gamma($\frac{1}{|q_j|}, 1$) distribution. Depending on the value of $q_j$, the cumulative distribution of $t$ is

$$F_j(t; \mu_j, \sigma_j, q_j) = \begin{cases} \frac{1}{\Gamma(1/q_j)} \int_0^{(\log t + \mu_j)/\sigma_j} y^{1/q_j - 1} \exp(-y) \mathrm{d}y & q_j > 0 \\ \Phi\left(\frac{\log t + \mu_j}{\sigma_j}\right) & q_j = 0 \\ 1 - \frac{1}{\Gamma(1/q_j)} \int_0^{(\log t + \mu_j)/\sigma_j} y^{-1/q_j - 1} \exp(-y) \mathrm{d}y & q_j < 0 \end{cases}$$

$F_j(t; \mu_j, \sigma_j, q_j)$ represents the probability of an entry being non-zero.

For simulation, first draw $\tilde{\boldsymbol{D}}_{i\cdot} \sim \mathcal{N}(0, \boldsymbol{\rho})$. $\tilde{Z}_{ij} = \mathbb{I}[\tilde{D}_{ij} > \Phi^{-1}(1 - F_j(M_i; \mu_{ij}, \sigma_j, q_j))]$ is the indicator of whether an entry is nonzero. To fill in the nonzero entries of taxon $j$, draw relative abundances from the generalized gamma distribution $f(t; \mu_{ij}, \sigma_j, q_j)$, then fill the values according to the ranking of $\tilde{D}_{ij}(i = 1, 2, \cdots, N)$. Given the library size $M_i$, we can simulate the counts for each taxon.

For each taxon $j$ in sample $i$, $\mu_{ij} = \mu_{0j} + X_i\gamma_j$. Taxon $j$ is differentially abundant if $\gamma_j \neq 0$. All the $\{\mu_{0j}, \sigma_j, q_j\}_{j=1,2,\cdots,P}$ are estimated based on a gut microbiome dataset from the Inflammatory Bowel Disease Multi-omics Database [12]. To control the signal noise ratio, I only include 50 taxa whose parameter estimate of $\mu$ is smaller than zero, $|q| < 10$ and $\sigma$ are among the smallest. To simulate a count matrix with 500 taxa, each set of parameters is applied to ten taxa. The correlation matrix of 500 taxa is block diagonal with ten identical blocks estimated from the original template data.

# References


[1] Tobin, J. Estimation of Relationships for Limited Dependent Variables. *Econometrica* **26**, 24 (1958).

[2] Amemiya, T. Regression Analysis when the Dependent Variable Is Truncated Normal. *Econometrica* **41**, 997–1016 (1973).

[3] Olsen, R. J. Note on the Uniqueness of the Maximum Likelihood Estimator for the Tobit Model. *Econometrica* **46**, 1211 (1978).

[4] Firth, D. Bias Reduction of Maximum Likelihood Estimates. *Biometrika* **80**, 27 (1993).

[5] Alam, T. F., Rahman, M. S. & Bari, W. On estimation for accelerated failure time models with small or rare event survival data. *BMC Medical Research Methodology* **22**, 169 (2022).

[6] Nocedal, J. & Wright, S. J. *Numerical Optimization* 2nd edn (Springer New York, NY, 2006).

[7] Ma, S. *et al.* A statistical model for describing and simulating microbial community profiles. *PLOS Computational Biology* **17**, e1008913 (2021).

[8] Human Microbiome Project Consortium. Structure, function and diversity of the healthy human microbiome. *Nature* **486**, 207–214 (2012).

[9] Schiffer, L. *et al.* HMP16SData: Efficient Access to the Human Microbiome Project Through Bioconductor. *American Journal of Epidemiology* **188**, 1023–1026 (2019).

[10] He, M., Zhao, N. & Satten, G. A. MIDASim: a fast and simple simulator for realistic microbiome data. *Microbiome* **12**, 135 (2024).

[11] Bonett, D. G. & Price, R. M. Inferential Methods for the Tetrachoric Correlation Coefficient. *Journal of Educational and Behavioral Statistics* **30**, 213–225 (2005).

[12] Lloyd-Price, J. *et al.* Multi-omics of the gut microbial ecosystem in inflammatory bowel diseases. *Nature* **569**, 655–662 (2019).

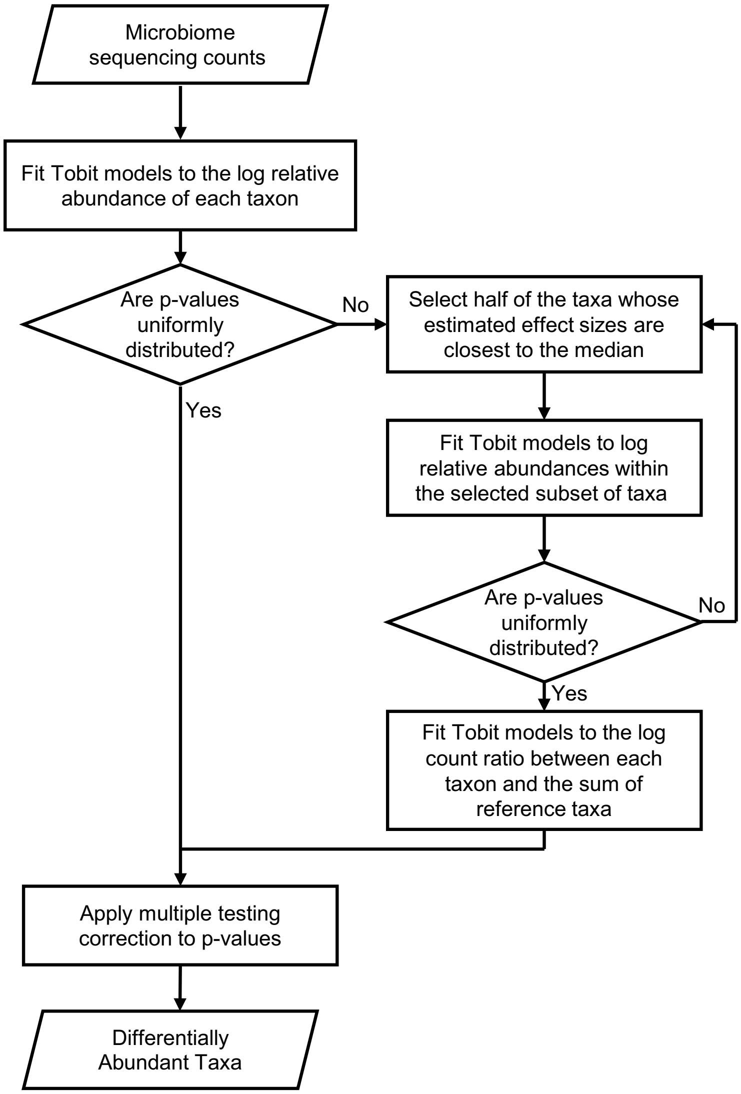


**Fig. S1**: **Analysis procedures of ADAPT.** The first step of ADAPT is to fit Tobit models to the log relative abundances of all the taxa. The second step is to find a subset of non-DA taxa as reference taxa. The third step is to find differentially abundant taxa by fitting Tobit models to log count ratios between individual taxa and the summed counts of reference taxa.

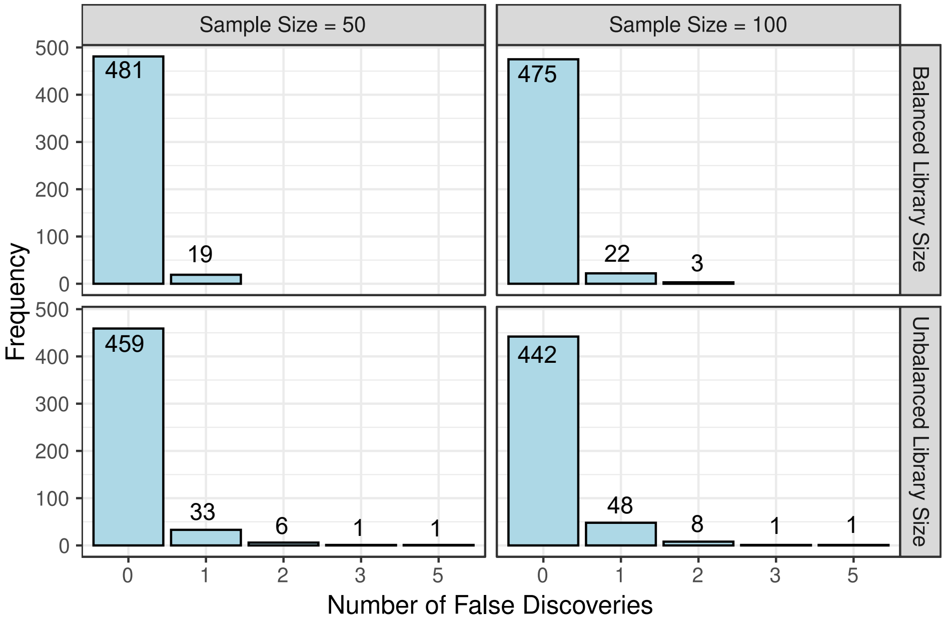


**Fig. S2**: **ADAPT's accuracy at confirming nonexistence of DA taxa under SparseDOSSA simulation framework.** ADAPT can correctly confirm that there are no DA taxa at over 95% accuracy if the library sizes are the same between two conditions. If the library sizes are significantly different between conditions, the accuracy drops to around 90%. When ADAPT accidentally confirms the existence of DA taxa, it rarely identifies more than one taxon as differentially abundant.

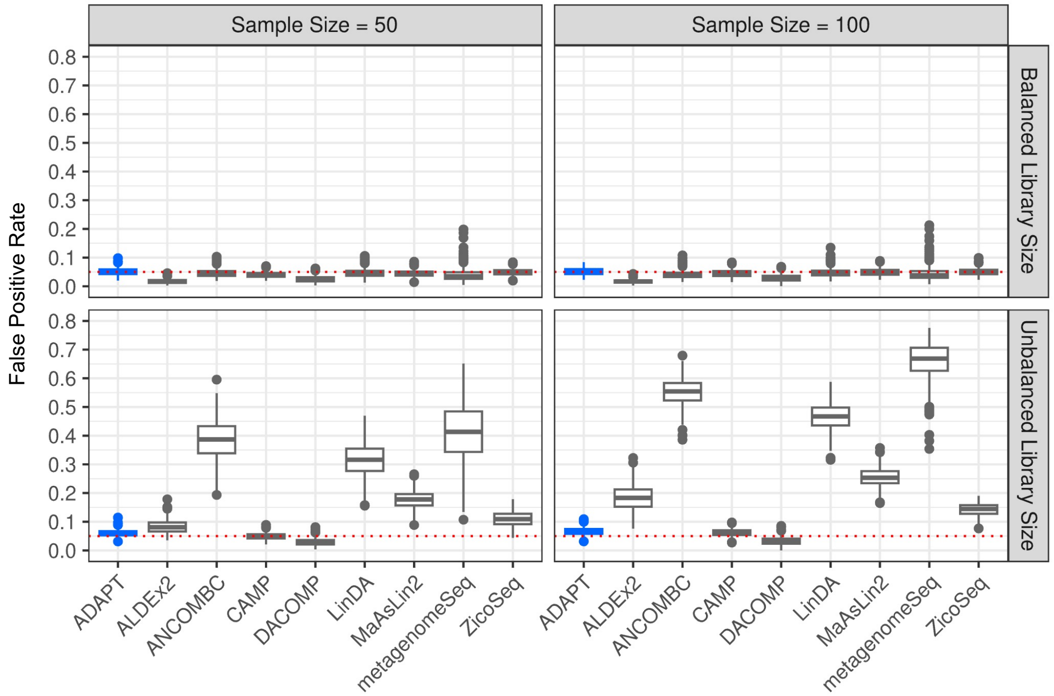


**Fig. S3**: **False positive rate comparison under MIDASim simulation framework.** Repeat simulations with no differentially abundant taxa following the same setting as Figure 1a in the main manuscript. The only difference is the simulation framework.

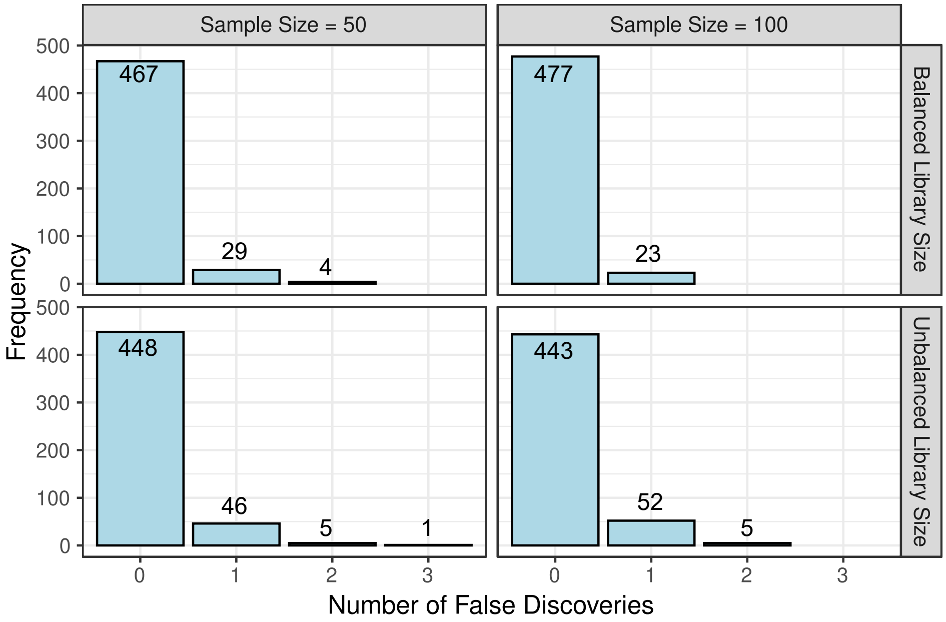


**Fig. S4**: **ADAPT's accuracy at confirming nonexistence of DA taxa under MIDASim simulation framework.** The performance is the same as the simulations under the SparseDOSSA simulation framework.

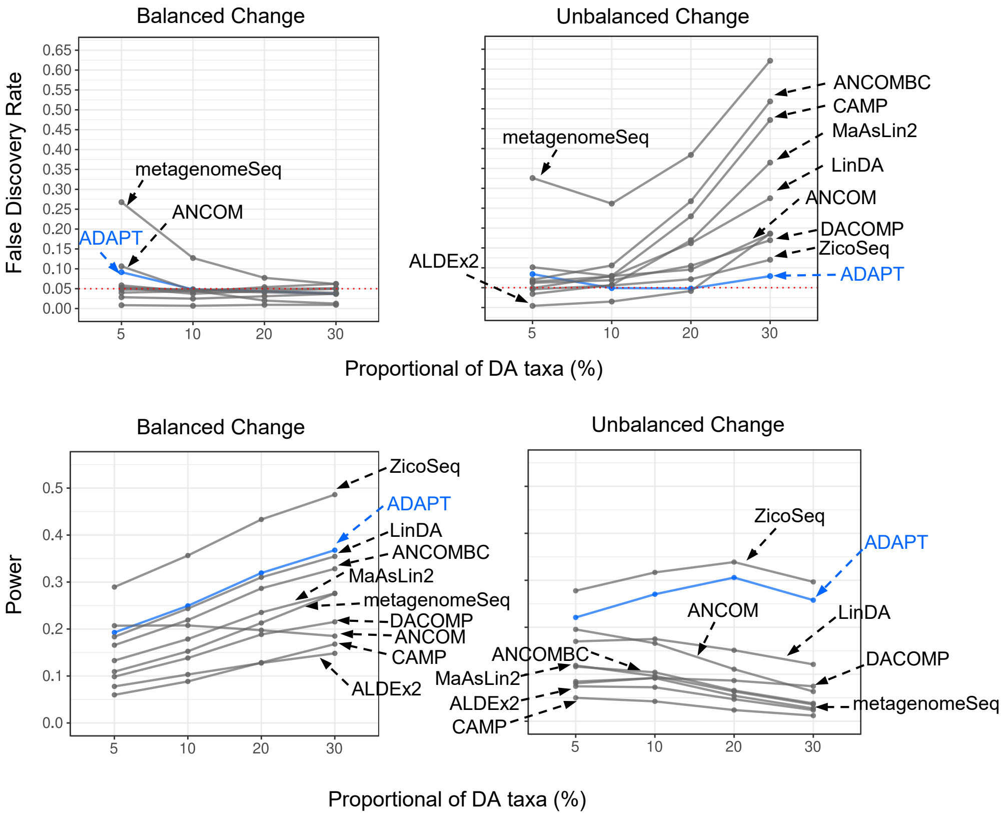


**Fig. S5**: **Simulation studies with different proportions of DA taxa based on the MIDASim framework.** The sample size is 100. The total number of taxa is 500. The proportion of DA taxa is 5%, 10%, 20%, or 30%. The average library size is $2 \times 10^4$ for both conditions. The average absolute abundance fold change of DA taxa is 4. The directions of absolute abundance changes of DA taxa may be balanced or unbalanced. This setting is the same as the one presented in Figure 2b of the main manuscript. The only difference is the simulation framework.

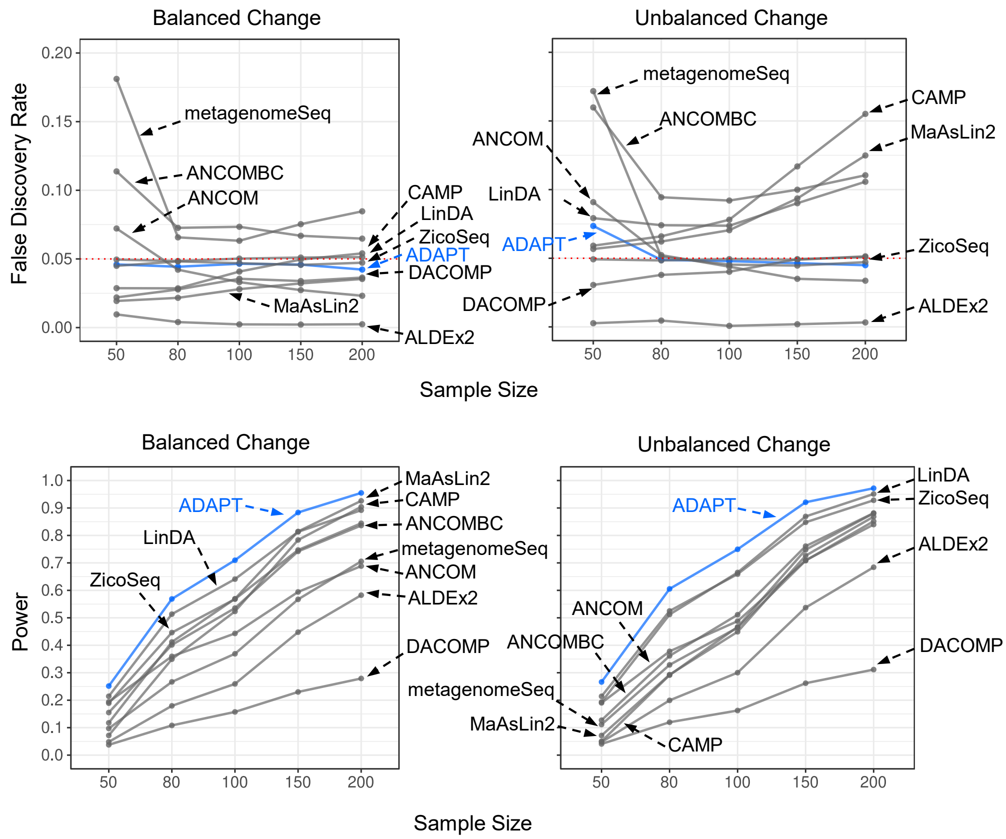


**Fig. S6**: **Simulation studies with different total sample sizes based on theSparseDOSSA framework.** The sample size is 50, 80, 100, 150, or 200. The total number of taxa is 500. The proportion of DA taxa is 10%. The average fold change is 5. The average library size is $2 \times 10^4$ for both conditions. The directions of absolute abundance changes of DA taxa may be balanced or unbalanced.

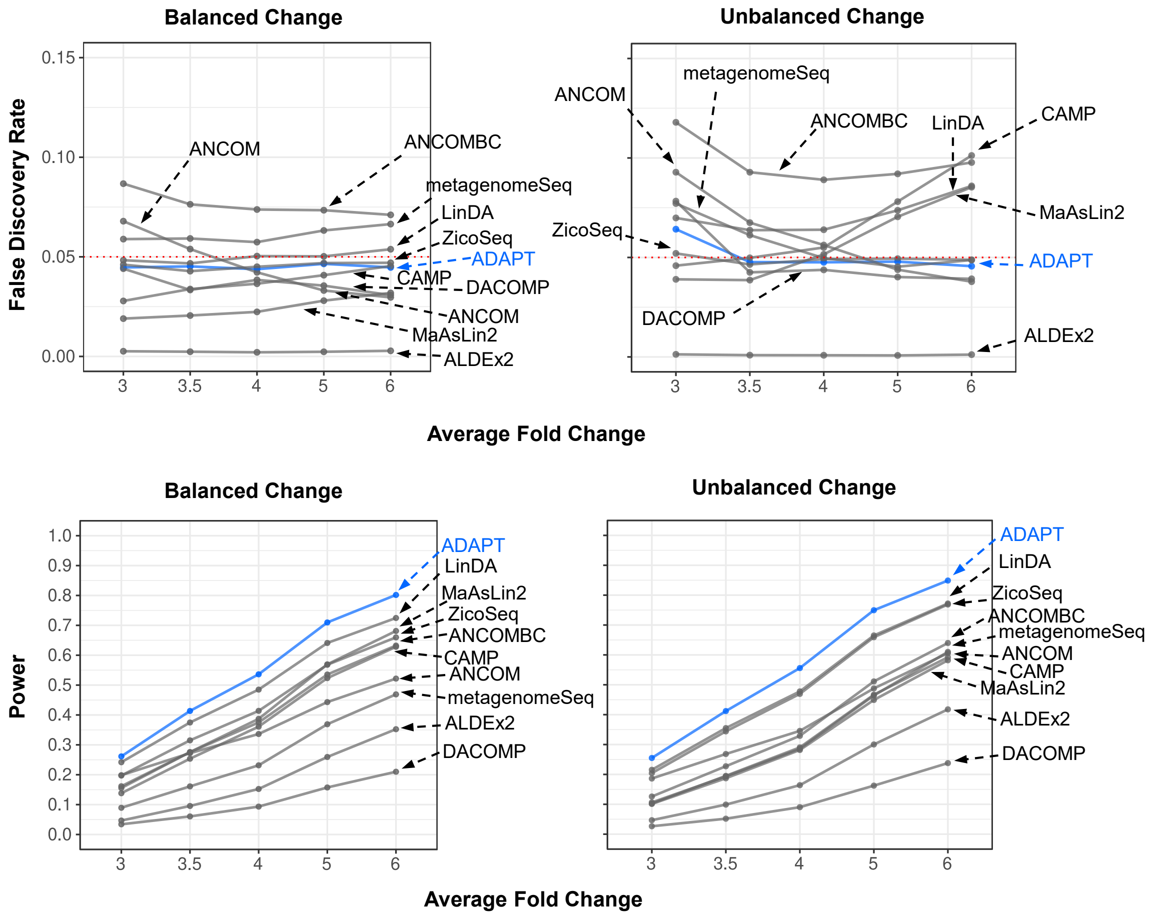


**Fig. S7**: **Simulation studies with different average fold changes of DA taxa based on the SparseDOSSA framework.** The sample size is 100. The total number of taxa is 500. The proportion of DA taxa is 10%. The average fold changes are 3, 3.5, 4, 5, or 6. The average library size is $2 \times 10^4$ for both conditions. The directions of absolute abundance changes of DA taxa may be balanced or unbalanced.

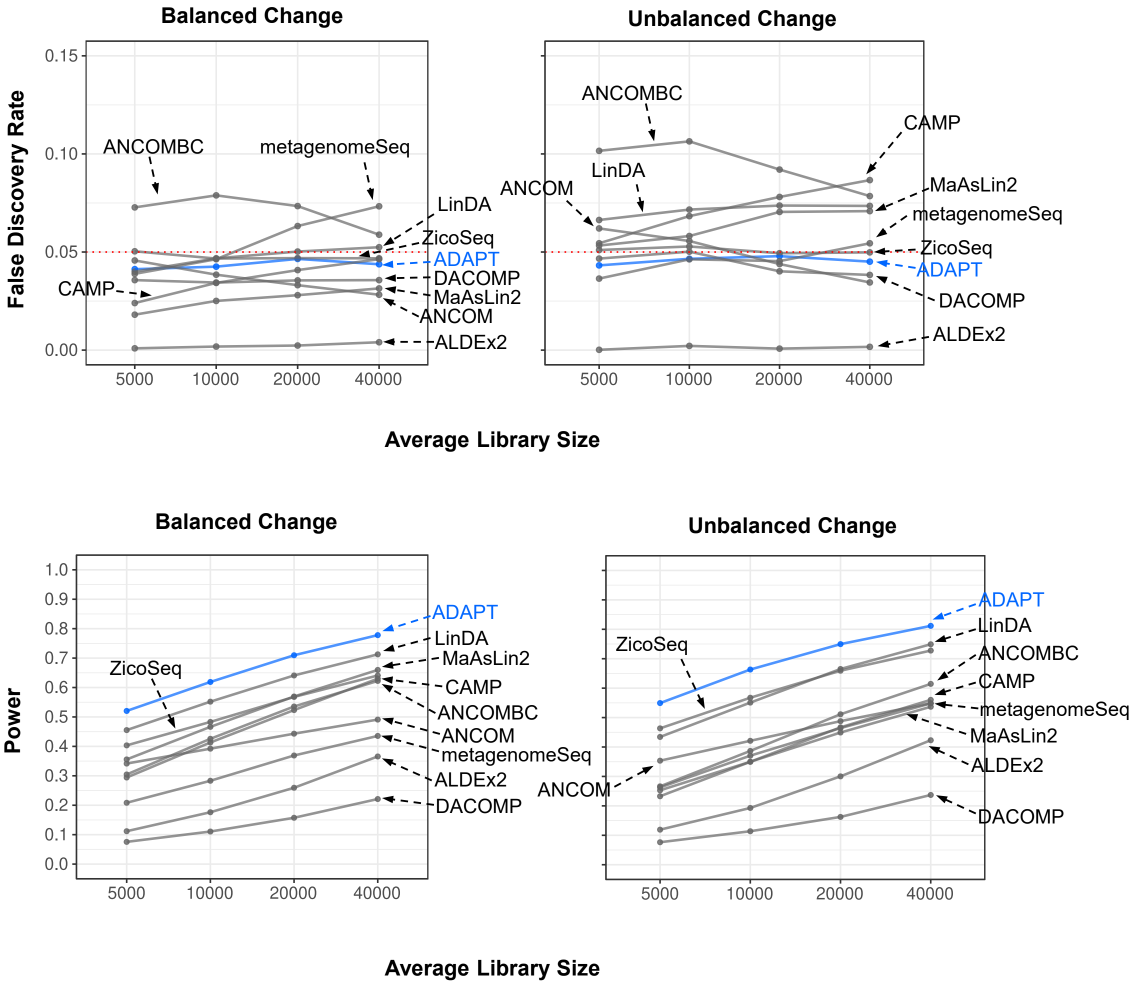


**Fig. S8**: **Simulation studies with different average library sizes based on theSparseDOSSA framework.** The sample size is 100. The total number of taxa is 500. The proportion of DA taxa is 10%. The average fold change is 5. The average library size is $5 \times 10^3$, $10^4$, $2 \times 10^4$, and $4 \times 10^4$ for both conditions. The directions of absolute abundance changes of DA taxa may be balanced or unbalanced.

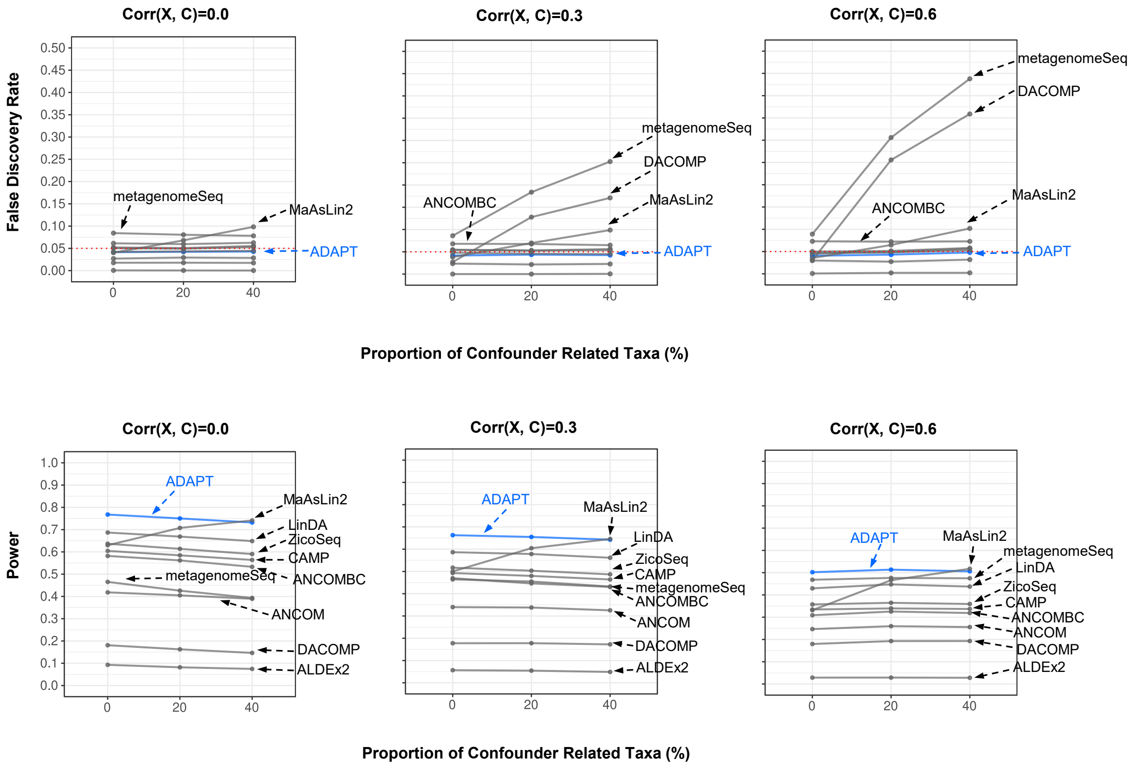


**Fig. S9**: **Simulation studies with potential confounders based on the SparseDOSSA framework.** The sample size is 100. The total number of taxa is 500. The proportion of DA taxa is 20%. The average library size is the same for both conditions at $2 \times 10^4$. The average absolute abundance fold change of DA taxa is 5. The proportion of taxa whose abundances correlate with the confounding variable is 0%, 20%, or 40%. The correlation between the binary variable and the continuous confounding variable is 0, 0.3, or 0.6. DACOMP and metagenomeSeq are the only two DAA methods that can not adjust for covariates.

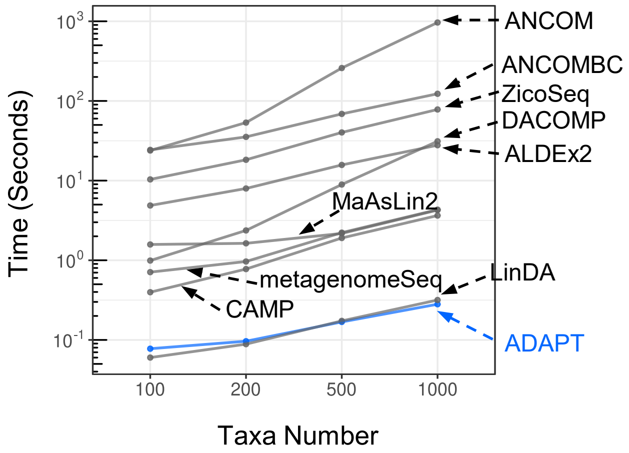


**Fig. S10**: **Computation time (seconds).** All simulations for time measurement have 100 samples and an average library size of $2 \times 10^4$. The total number of taxa is 100, 200, 500, or 1000. 10% of all the taxa are DA. We generate 500 replicates for each simulation setting and report all DAA methods' mean computation time. Each method is allocated four cores (Intel Xeon Gold 6154) and 16GB of memory.

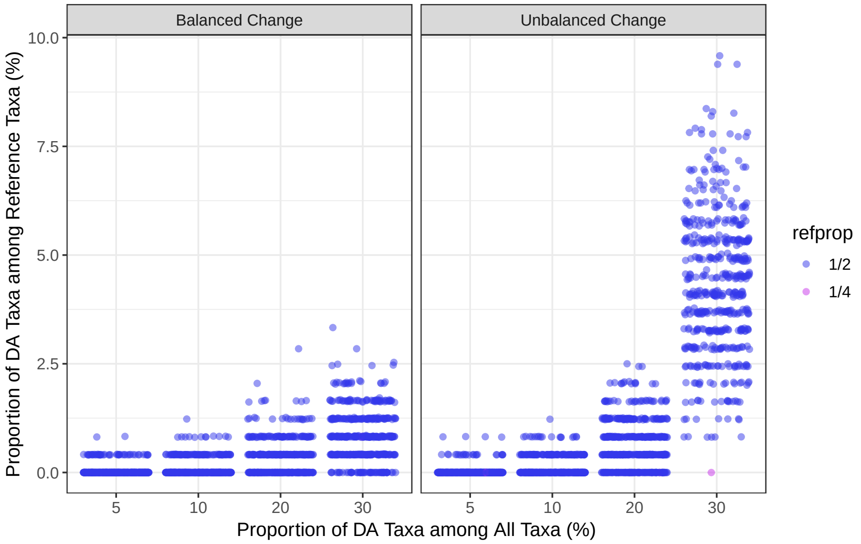


**Fig. S11**: **Contamination of DA taxa in the reference taxa set**. Contamination refers to the proportion of DA taxa among the reference taxa selected by ADAPT. Each point corresponds to a simulation replicate under the SparseDOSSA framework. Different colors indicate whether ADAPT select one half or one fourth of all the taxa as reference.

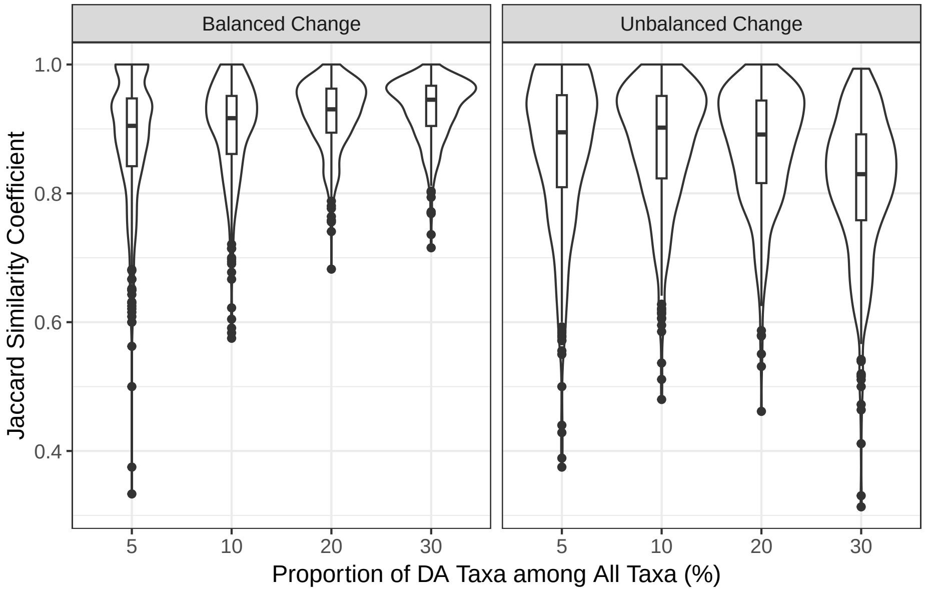


**Fig. S12**: **Overlap of detected DA taxa by ADAPT and the detected DA taxa by choosing all the nonDA taxa as reference.** The simulations are based on the SparseDOSSA framework. The overlap is evaluated by calculating the Jaccard similarity coefficient (also called "intersection over union").

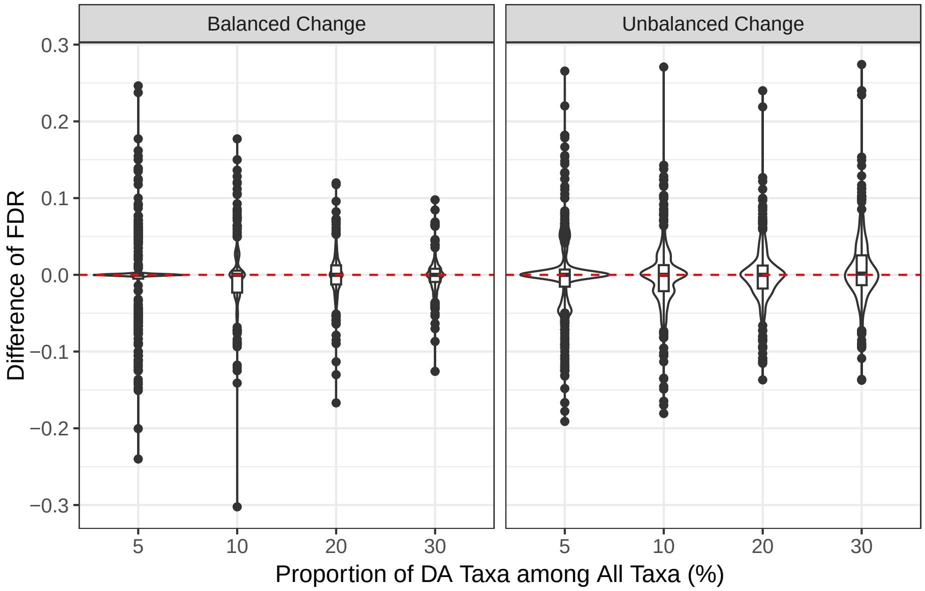


**Fig. S13**: **Difference of false discovery rates between detected DA taxa by ADAPT and the detected DA taxa by choosing all the nonDA taxa as reference.** The simulations are based on the SparseDOSSA simulation framework.

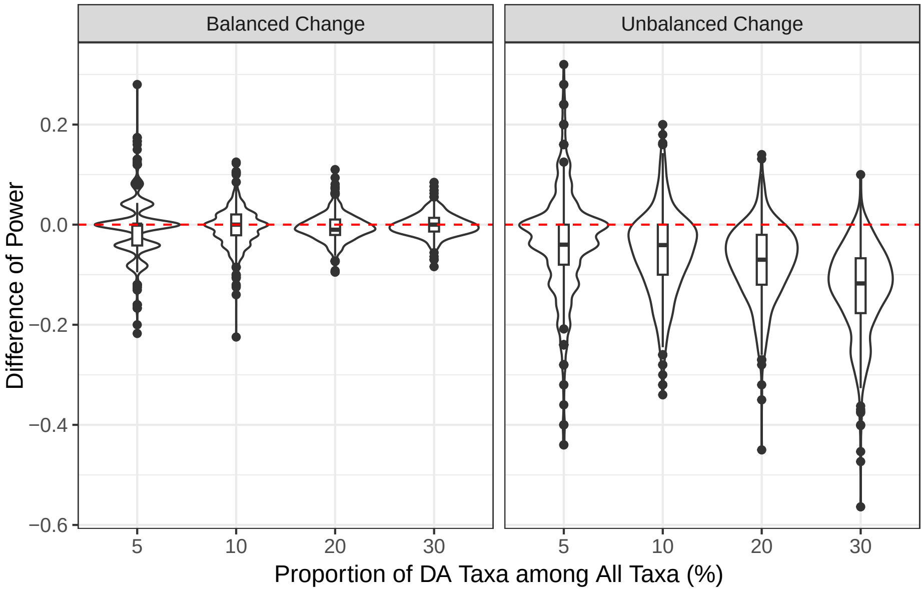


**Fig. S14**: **Difference of power between detected DA taxa by ADAPT and the detected DA taxa by choosing all the nonDA taxa as reference.** The simulations are based on SparseDOSSA simulation framework.
